# Normalization of muscle strength measures in barbell exercises by sex, age, and anthropometry: notes on allometry

**DOI:** 10.1038/s41598-026-60646-z

**Published:** 2026-07-06

**Authors:** Eduard Isenmann, Stephan Geisler, Tim Havers, Simon Gavanda, Patrick Diel, Steffen Held, Ulrich Flenker

**Affiliations:** 1 Department of Fitness and Health, IST University of Applied Sciences, Duesseldorf, Germany; 2 Department of Molecular and Cellular Sports Medicine, Institute of Cardiovascular Research and Sports Medicine, German Sport University Cologne, Cologne, Germany; 3 Exercise Biology Group, School of Medicine and Health, Technical University of Munich, Munich, Germany; 4 Department of Sport and Management, IST University of Applied Sciences, Duesseldorf, Germany

**Keywords:** Maximal strength, Allometric scaling, Strength classification, Performance, Framework, Anatomy, Health care, Medical research, Physiology

## Abstract

Accurate strength assessment requires consideration of individual anthropometric and demographic differences. Traditional absolute and body mass-normalized measures often fail to capture inter-individual variability, thereby limiting the precision of strength testing and classification. This study aimed to investigate the influence of age, body mass, height, and anthropometric characteristics on strength capacity and to systematically integrate these variables into a classification framework. A total of 393 individuals (197 females, 196 males), aged 16–64 years and ranging from beginners to elite powerlifters, were recruited. One-repetition maximum (1-RM) strength was assessed in the bench press (BP), back squat (SQ), and deadlift (DL). Age, height, body mass, and selected anthropometric variables including arm and leg lengths, were recorded. Multiple regression analyses were used to quantify the influence of these variables on 1-RM performance. Based on significant predictors, individualized z-scores were computed to standardize 1-RM values relative to relevant anthropometric and demographic factors. The applicability of these z-scores to interval-based classification frameworks was evaluated. Age, body mass, and height were identified as significant determinants of maximal strength across all 3 exercises. Incorporation of these variables into z-score calculation enabled a more precise and individualized assessment of strength performance. Attempts to apply z-scores to interval-based classification frameworks revealed substantial within-category heterogeneity, indicating that such frameworks are unsuitable for accurate strength classification. This study establishes an anthropometry- and demography-adjusted framework for strength assessment in BP, SQ, and DL among healthy adults aged 16–64 years. Standardized z-scores provide a biologically meaningful alternative to conventional normalization methods. The proposed z-score framework provides a reproducible, data-driven tool for individualized strength assessment in the BP, SQ and DL, with direct applications in sport science research, performance monitoring, and strength-based rehabilitation for healthy adults aged 16–64 years.

**Supplementary Information:**

The online version contains supplementary material available at 10.1038/s41598-026-60646-z.

## Introduction

Precise performance assessment is fundamental to sports science, providing essential insights for training management, talent identification, and injury prevention^1^. Across disciplines, standardized testing protocols and reference values are essential to ensure reproducibility and enable meaningful comparisons^1–6^. While endurance testing benefits from widely accepted metrics and normative frameworks^7–12^, strength testing remains comparatively underdeveloped in this regard^13^.

Multiple valid methods for assessing maximal strength exist, of which the one-repetition maximum (1-RM) test is among the most widely used^13,14^. Although standardized 1-RM protocols are available^15^, the interpretation of raw strength values remains limited because individual characteristics, including anthropometrics, sex, and age, substantially influence performance outcomes^16–20^. Even small variations in exercise execution, such as joint angles range of motion, barbell placement or rest period can introduce considerable discrepancies in performance outcomes^19,21–23^. These factors complicate cross-individual and cross-study comparisons and highlight the need for a normalization framework that accounts for biomechanical variability^24,25^.

Reference value concepts, widely applied in clinical diagnostics, offer a promising solution^26,27^. By defining a reference population based on clear selection criteria, collecting data under standardized conditions, and deriving percentile-based normative values, individual performances can be evaluated relative to population-based reference ranges^28^. Within endurance-oriented performance assessment, methodologies such as $\:\dot{V}{\mathrm{O}}_{2}\mathrm{max}$ percentiles, lactate thresholds, and critical power models enable meaningful comparisons across athletes^29,30^. In contrast, strength-oriented assessments currently lack a universally accepted reference framework^31,32^. Most assessments still rely on absolute 1-RM values or simple relative strength ratios (e.g., strength normalized to body mass), which do not fully account for the biomechanical impact of anthropometric variability^33^. Consequently, existing approaches remain largely theoretical and insufficiently supported by empirical data, particularly for elite athletes whose morphological and physiological profiles differ substantially from general populations^34–36^.

The primary aim of this study is to examine the influence of age, body mass, and anthropometric characteristics across both biological sexes to establish normative strength references for healthy individuals. We focus specifically on 3 fundamental barbell exercises: the bench press (BP), back squat (SQ) and deadlift (DL). To achieve this aim, we propose an anthropometry-adjusted strength classification model based on multiple regression analysis, incorporating key morphological variables such as height, limb lengths, and body mass^37–39^. By integrating these factors into predictive models, expected strength levels can be estimated and expressed as standardized z-scores, enabling more precise comparisons across populations and performance levels. We hypothesize that anthropometric factors significantly influence strength outcomes and that predictive models incorporating these variables will demonstrate higher validity than conventional strength assessment methods. Furthermore, this reference-based approach is expected to identify systematic performance deviations, highlighting individuals with exceptional strength capacities relative to their anthropometric profiles. The potential impact of this study extends beyond academic research. A validated reference system could enhance talent identification, support individualized training programming, and improve longitudinal athlete monitoring by enabling early detection of performance plateaus or atypical strength developments. By developing a reproducible, scalable, and scientifically robust classification framework, this study aims to advance strength diagnostics toward a more objective, individualized, and broadly applicable tool across diverse populations and performance levels.

## Methods

### Data acquisition

#### Participants

A priori sample size calculation was conducted to ensure sufficient statistical power to detect meaningful relationships between anthropometric factors and strength performance outcomes (i.e. 1-RM). Given the use of multiple linear regression analysis, the required sample size was determined using Cohen’s *f²* effect size framework and power analysis calculations based on previous studies examining 1-RM and anthropometry^33,40^. The following parameters were considered: effect sizes of 0.02 (small), 0.15 (medium), and 0.35 (large)^41^; a significance level (*α*) of 0.05; statistical power (1-*β*) of 0.80; and up to 6 predictors, including body mass, height, arm and limb lengths, sex, and age. Using G*Power 3.1.9.7, power analysis for multiple linear regression (fixed model, *R²* increase) indicated that to detect a small effect (*f²* = 0.02), at least 395 participants would be required; for a medium effect (*f²* = 0.15), 88 participants; and for a large effect (*f²* = 0.35), 39 participants^42^. Given the influence of multiple anthropometric and biomechanical factors, the study aimed to detect at least a medium effect (*f²* = 0.15) with sufficient precision across sex and performance subgroups. Therefore, a minimum total sample size of *n* = 250 participants was targeted to allow for robust subgroup analyses and accommodate potential attrition or missing data.

Participants were recruited across a wide range of performance levels to enhance generalizability. This included absolute beginners who had never or only irregularly performed the tested movements, as well as elite athletes who regularly compete at national level or qualify for international competitions. Participants were stratified across age and body composition ranges to enhance generalizability. All participants were required to demonstrate technical proficiency in each exercise prior to participation, as assessed by experienced, trained personnel with expertise in resistance exercise assessment. Participants were required to demonstrate the ability to perform each exercise through the full range of motion with controlled technique before being admitted to 1-RM testing. Training experience was assessed; however, it was not included in the analyses, as it did not allow for meaningful differentiation of performance levels. Self-reported training experience did not reliably reflect actual strength capacity. Data were collected on general resistance training experience, exercise-specific experience, weekly training frequency of the tested exercises, and the use of assistance exercises. Nevertheless, these variables were not further considered, as they did not provide a valid or consistent basis for classifying participants according to performance level.

Exclusion criteria included existing musculoskeletal injuries, ongoing medical treatment, the use of substances included on the World Anti-Doping Agency (WADA) Prohibited List. All participants provided written informed consent prior to the commencement of the study, confirming their voluntary participation. For participants under the age of 18 years, written consent was additionally obtained from a parent or legal guardian. This study was approved by the local ethics committee of the IST University of Applied Sciences (172025IST233) and conducted in accordance with the Declaration of Helsinki^43^.

#### Study protocol

The 1-RM in BP, SQ, and DL was assessed in a cohort ranging from beginners to elite powerlifters. Testing was conducted between 3:00 PM and 7:00 PM to minimize the influence of circadian rhythm on muscular strength. Participants were instructed to refrain from intense exercise 48 h prior and to avoid all exercise 24 h before testing. Dietary habits were maintained, with the last meal consumed at least 2 h before testing; immediate pre-test food intake was prohibited. The use of pre-workout boosters, or caffeine tablets was strictly forbidden, whereas habitual creatine supplementation and moderate coffee consumption (e.g., a standard cup of coffee or espresso) were permitted if part of the participant’s routine and not consumed for acute ergogenic purposes.

#### Performance testing

1-RM in the BP, SQ and DL was assessed following the guidelines of the National Strength and Conditioning Association (NSCA)^15^. To minimize potential fatigue effects between exercises, testing was split into 2 sessions scheduled at least 72 h apart: SQ and BP on day 1, and DL on day 2. All assessments used standardized competition equipment. A 20 kg standardized competition barbell (Eleiko SPORT GmbH, Herrieden, Germany) was used for all male exercises and for the female SQ and DL, while a 15 kg barbell was used for the female BP. Weight increments utilized competition plates (25 kg to 10 kg) with a standardized 22.5 cm radius. The 1-RM was determined within 4 to 8 attempts, with incremental steps of 5–20 kg (SQ/DL) and 2.5–10 kg (BP). The smallest allowable increment was 1 kg. If a lift was unsuccessful, participants were allowed to repeat the attempt or reduce the load for a subsequent attempt.

A standardized warm-up and testing protocol was implemented for all participants. The general warm-up consisted of 10 min of low-to-moderate intensity aerobic activity (rowing ergometer, cross-trainer, or treadmill locomotion) performed at a self-selected pace that did not induce substantial fatigue. This was followed by an task-specific warm-up comprising 3–4 sets, depending on individual needs. Participants were generally advised to perform 3 preparatory sets at approximately 50% of the estimated 1-RM for 10 repetitions, 70% for 5 repetitions, and 80% for 3 repetitions. Movement tempo during warm-up sets was controlled and technically deliberate, without intentional slowing. Rest intervals were standardized to 2 min between warm-up sets and 3 min between the final warm-up set and the first maximal attempt.

The first 1-RM attempt was performed at approximately 90% of the estimated 1-RM. Subsequent maximal attempts were separated by 4-minute rest intervals to minimize fatigue effects. The protocol aimed to determine the individual 1-RM within a maximum of 5 attempts whenever possible.

All maximal attempts were conducted under the supervision of experienced spotters to ensure participant safety. Verbal encouragement was provided consistently during maximal efforts. A lift was considered valid only if predefined technical criteria were clearly met upon visual inspection. For the SQ performed in either high-bar or low-bar position according to individual preference, the hip crease had to descend below the superior aspect of the knee joint.t. In the BP, the barbell was required to come to a brief, controlled pause on the chest before the concentric phase. For the DL, full extension of the hip and knee joints (lockout) had to be achieved with an upright torso position. Attempts were deemed invalid if these criteria were not fulfilled, if there was a loss of control of the load, or if external assistance from the spotters influenced the lift.

The use of weightlifting belts, wrist wraps, knee sleeves, and chalk was permitted to ensure ecological validity; however, equipment such as bench shirts, deadlift suits, or lifting straps was prohibited. For the DL, both conventional and sumo techniques were allowed to accommodate individual biomechanical preferences. In the BP, a bridging technique was permitted providing the participants’ gluteal muscles maintained contact with the bench.

#### Anthropometry

To quantify the influence of anthropometric factors on 1-RM, several key morphological parameters were recorded alongside chronological age.

Body mass was measured using a calibrated digital scale (Etekcity EB4074C, Anaheim, CA, United States of America), with participants wearing lightweight sportswear (T-shirt and shorts) and no shoes.

Body height and segmental anthropometry were measured using a stadiometer (Seca 201, Hamburg, Germany). Height was measured with participants standing upright against a wall, barefoot, with heels together and head in a neutral anatomical position. The arm length was measured from the acromion to the midpoint between the thumb and index finger (measured above the arm), with the arm extended. The femur length was measured from the greater trochanter to the lateral knee gap (laterally along the femur). Additionally, barbell displacement from the start to the end position of the deadlift was measured. Due to differences in lifting technique, substantial variations in movement distance could have been expected. However, the observed differences were minimal and were therefore not considered further in the analysis.

#### Allometry

Allometry is a pervasive phenomenon in biology. It is present when variables *x* and *y* were linked *via*

1 $$\:y\hspace{0.17em}=\hspace{0.17em}a\:xb$$

or, equivalently,

2 $$\:log\left(y\right)\hspace{0.17em}=\hspace{0.17em}log\left(a\right)\hspace{0.17em}+\hspace{0.17em}b\:log\left(x\right)$$

Constant *b* is termed “scaling factor”.

The concept was coined by the finding that basal metabolic rates *I* relate to body masss accordingly. The relation

3 $$\:log\left(I\right)\hspace{0.17em}=\hspace{0.17em}log\left(I0\right)\:+\:^{\frac{3}{4}}\:log\left(m\right)$$

is known as Kleiber’s Law^44^. The scaling factor 3/4 is commonly explained by surface to volume ratios. Larger and heavier animals show higher values of *I*. But their reduced surface to volume ratios is thought to reduce thermal losses disproportionally. Well before Kleiber’s work, Snell had proposed a factor of 2/3^45^.

Allometry as such is undisputed. But there is considerable controversy concerning the true nature of the phenomenon^46^ and the scaling factors^47^.

### Data analysis

Anthropometric measures served as independent variables in statistical models for each combination of exercise ($\:E$ = BP, SQ, DL) and sex (female, F; male, M). The 1-RM of the respective exercises always represented the dependent variable.

Multiple linear regression were used for statistical modeling. Initial model fitting assumed linear relationships between predictors and 1-RM. To account for allometric scaling effects, both 1-RM and body mass were systematically log-transformed prior to analysis.

Fulfilment of model assumptions, namely Gaussianity and homoscedasticity, was evaluated using diagnostic plots. Gaussianity was assessed using normal quantile-quantile plots, while homoskedasticity was examined via residual plots. Potential non-linearities were evaluated using partial residual plots. When noteworthy curvature was observed, the respective variable was incorporated into the model using second- or third-order polynomials.

Residuals $$\:R\left(E\right)$$ were calculated as the differences between observed and model-predicted performances resulting from the model fitted to $$\:E$$. The residual standard deviation *RSD (E)* was determined for each task-specific model. Because regression residuals have a mean of zero by definition, standardization using the division $R(*E*)/*RSD(E)* allowed for transformation into z-scores, approximately scaling to −3 < *z*_*i*_*(m)* < + 3.

Gaussianity of the residuals is a fundamental model assumption. Residuals derived from models fitted separately for female and male athletes therefore constitute a multivariate Gaussian distribution. This enables calculation for Mahalanobis distances ($\:{D}_{M}^{2}$). Values representing identical cumulative probabilities feature identical values of $\:{D}_{M}^{2}$. Ostensibly, they fall on the same ellipsoid representative of a given probability. Values of $\:{D}_{M}^{2}$ resulting from $\:k$ gaussians ideally follow a $\:{\chi\:}^{2}$-distribution with $\:k$ degrees of freedom ($\:{D}_{M}^{2}\sim\:{\chi\:}_{k}^{2}$).

This statistical framework allows identification of individuals exhibiting unusual combinations of strength capacity by defining critical thresholds of $$$\:{D}_{M}^{2}$$.

All analyses were performed using R (R Core Team 2022).

### Development of an overlapping interval-based performance classification

To evaluate the applicability of the derived z-scores within a categorical framework, an additional overlapping, interval-based performance classification was implemented. Seven performance levels were defined: untrained, beginner, intermediate, trained, advanced, highly advanced, and elite; based on established frameworks and terminology, without incorporating information on training history or training behavior^34,35^. Category boundaries were derived from the empirical distribution of the study sample, with thresholds set at approximately half-integer intervals along the z-score continuum (e.g., < −1.4 for untrained, − 1.6 to − 0.4 for beginner, − 0.6 to 0.6 for intermediate). To account for the continuous nature of performance and to minimize artificial discontinuities between adjacent categories, overlapping ranges were introduced by extending each boundary by ± 0.1 z-score units. For each category, corresponding percentile ranges, mean absolute 1-RM values, and mean relative strength ratios (1-RM/body mass) were calculated separately for each exercise and sex. This classification was intended as a supplementary descriptive framework and is presented alongside the continuous z-score approach to better capture and illustrate the heterogeneity within ordinal performance categories.

## Results

### Descriptive data

A total of 393 participants (197 females and 196 males) were included in the study. Participants with no prior experience in performing 1-RM tests in the BP, SQ or DL, as well as elite powerlifters who compete at national and international levels, including European and World Championships, were tested. The cohort had a mean age of 27.8 ± 8.3 years (range: 16 to 64 years). Mean height was 174.1 ± 10.3 cm (range: 150–206 cm), with men averaging 180.8 ± 6.8 cm and women 167.8 ± 6.6 cm. Mean body mass was 76.5 ± 14.8 kg (range: 46–124 kg), with men averaging 85.2 ± 11.3 kg and women 67.3 ± 9.6 kg. Detailed distributions of age, body mass, and height are provided in the supplemental material (S1-3).

### Model formulae

To predict an individual’s performance *P*_*i*_*(E)* for exercise *E* and individual *i*, the following general equation was used:

$$\:{P}_{i}\left(E\right)=I\left(E\right)+\sum\:{c}_{j}\left(E\right)\cdot\:{M}_{ij},$$

where $\:I\left(E\right)$ denotes the task-specific intercept, $\:{c}_{j}\left(E\right)$ are the coefficients for the measured variables *j* (e.g., arm length *l*_*a*_, femur length *l*_*f*_, body mass etc.), and $\:{M}_{ij}$ represents the corresponding measurement or its algebraically transformed value (e.g., logarithmic or power transformation).

The variable $\:E$ refers to the 1-RM values for the BP, SQ and DL, which were analyzed separately for female and male participants and using either the complete or reduced model versions.

To standardize individual performance, a performance score *Z*_*i*_*(E)* was calculated for each individual $\:i$ and exercise combination $\:E$:

$$\:{Z}_{i}\left(E\right)=\frac{{F}_{i}\left(E\right)-{P}_{i}\left(E\right)}{RSD\left(E\right)}=\frac{{R}_{i}\left(E\right)}{RSD\left(E\right)},$$

where $\:F$_*i*_*(E)* is the observed 1-RM performance, *R*_*i*_*(E)* is the residual (i.e., the difference between observed and predicted performance), and $\:RSD$ for indicated model. This approach enables direct comparison of strength levels across individuals while accounting for anthropometric factors and exercise type. The resulting z-scores provide a more precise classification of performance relative to the expected strength given an individual’s body structure.

### Model coefficients and residual standard deviations

The regression models estimate maximal 1-RM based on anthropometric variables. The resulting complete models for females (Table 1) and males (Table 2) include body mass (log(m)), height (h), age (a), and for females additionally arm length (la) and femur length (lf) in the SQ and DL models. Partial residual plots of the complete allometric model are provided in the supplementary material (S4–S9).

The consistently reduced models (Tables 1 and 2) simplify the approach by removing less relevant predictors while maintaining similar accuracy. Across all models, body mass is the strongest predictor, whereas height, age, and limb segment lengths exhibit smaller, exercise-dependent effects. RSDs indicate slightly better predictive accuracy for the complete models, particularly for the DL (S10, S11).

**Table 1:** Coefficients of the models fitted to the performances of female individuals.

M_j_	Complete model			Reduced model		
	BP 1-RM	SQ 1-RM	DL 1-RM	BP 1-RM	SQ 1-RM	DL 1-RM
Intercept	−40.65	−26.66	−25.41	−41.41	−32.84	−32.99
l_a_	−0.01289	–	–	ni	ni	ni
l_f_	–	−0.02588	−0.02772	ni	ni	ni
h	−0.01345	−0.0137	–	−0.01937	−0.02515	−0.01657
a	0.03602	0.1376	0.1245	0.04317	0.03247	0.05272
a^2^	−0.00063	−0.00392	−0.00326	−0.00073	−0.00068	−0.00092
a^3^	–	3e-05	2e-05	ni	ni	ni
log(m)	21.37	14.54	13.56	21.74	18.26	17.86
log(m)^2^	−2.42	−1.586	−1.495	−2.455	−2.016	−2.002

1-RM = one repetition maximum, a = age, BP = bench press, DL = deadlift, h = height, la = arm length, lf = femur length, m = body mass, ni = not included, SQ = squat.

**Table 2:** Coefficients of the models fitted to the performances of male individuals.

M_j_	Complete model			Reduced model		
	BP 1-RM	SQ 1-RM	DL 1-RM	BP 1-RM	SQ 1-RM	DL 1-RM
Intercept	−26.23	−30.03	−34.96	−26.23	−29.93	−33.51
l_a_	–	–	–	ni	ni	ni
l_f_	–	–	–	ni	ni	ni
h	−0.0158	−0.02404	−0.00999	−0.0158	−0.02408	−0.01987
a	0.00576	–	–	0.00576	−0.00105	0.00137
a^2^	−0.00018	−0.00017	–	−0.00018	−0.00015	−0.00015
a^3^	–	–	–	ni	ni	ni
log(m)	13.62	15.79	17.9	13.62	15.76	17.43
log(m)^2^	−1.357	−1.558	−1.867	−1.357	−1.554	−1.781

1-RM = one repetition maximum, a = age, BP = bench press, DL = deadlift, h = height, la = arm length, lf = femur length, m = body mass, ni = not included, SQ = squat.

### Allometry

All models exhibited significant curvatures of the partial relation between log-transformed strength performances *log(1RM)* and log-transformed body mass *log(m)*. Both, first order *log(m)* and second order terms *log(m)*$\:{}^{2}$, are statistically significant in all cases (*p* < 0.05). The partial residual plots demonstrate the appropriateness of 2nd order relations. See supplemental material, figures S5 – S15.

**Fig. 1:**
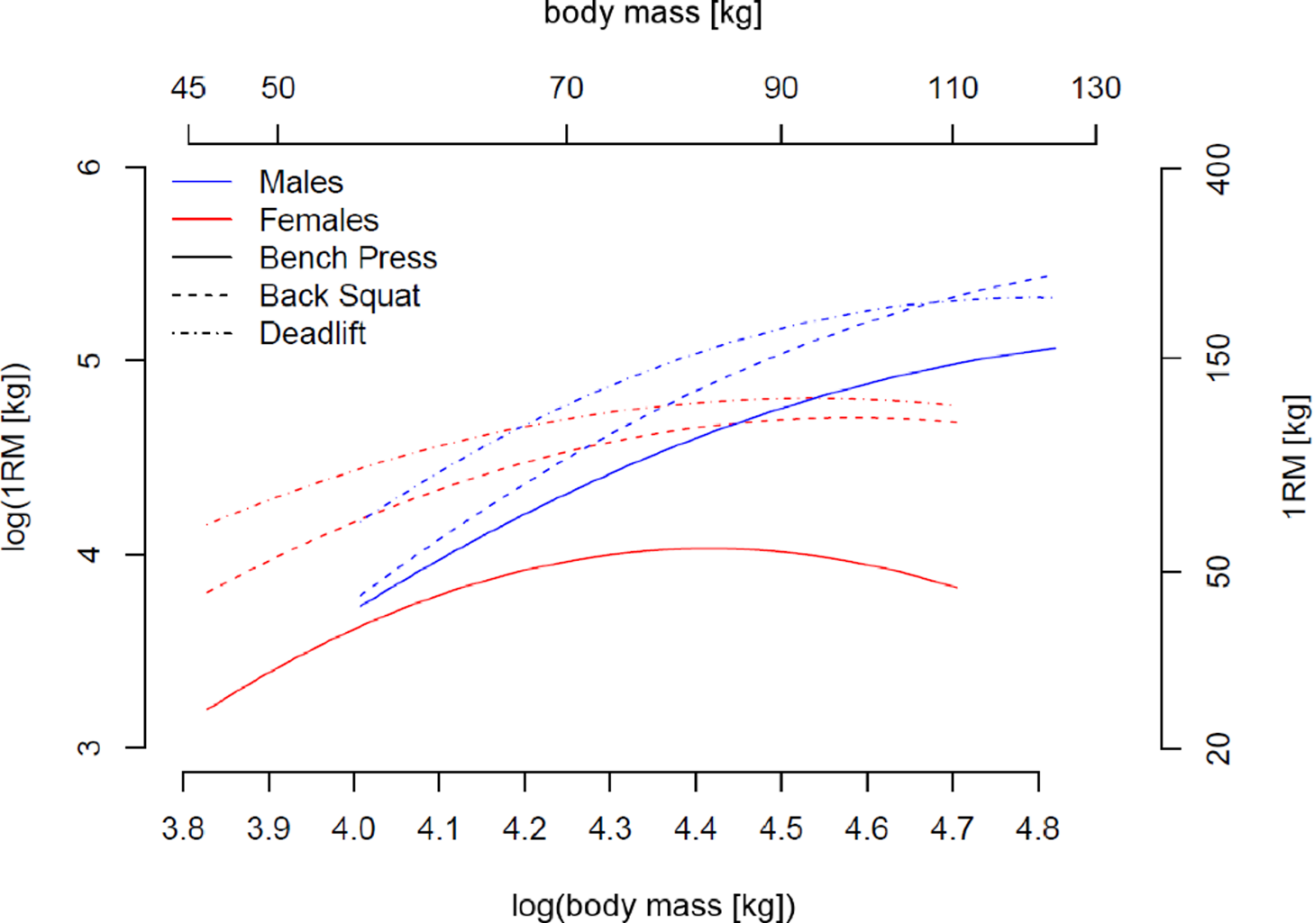
Partial 2nd order regression plots of log-transformed strength performances vs. body mass (full models). All other independent variables have been set to the respective group medians.

**Fig. 2:**
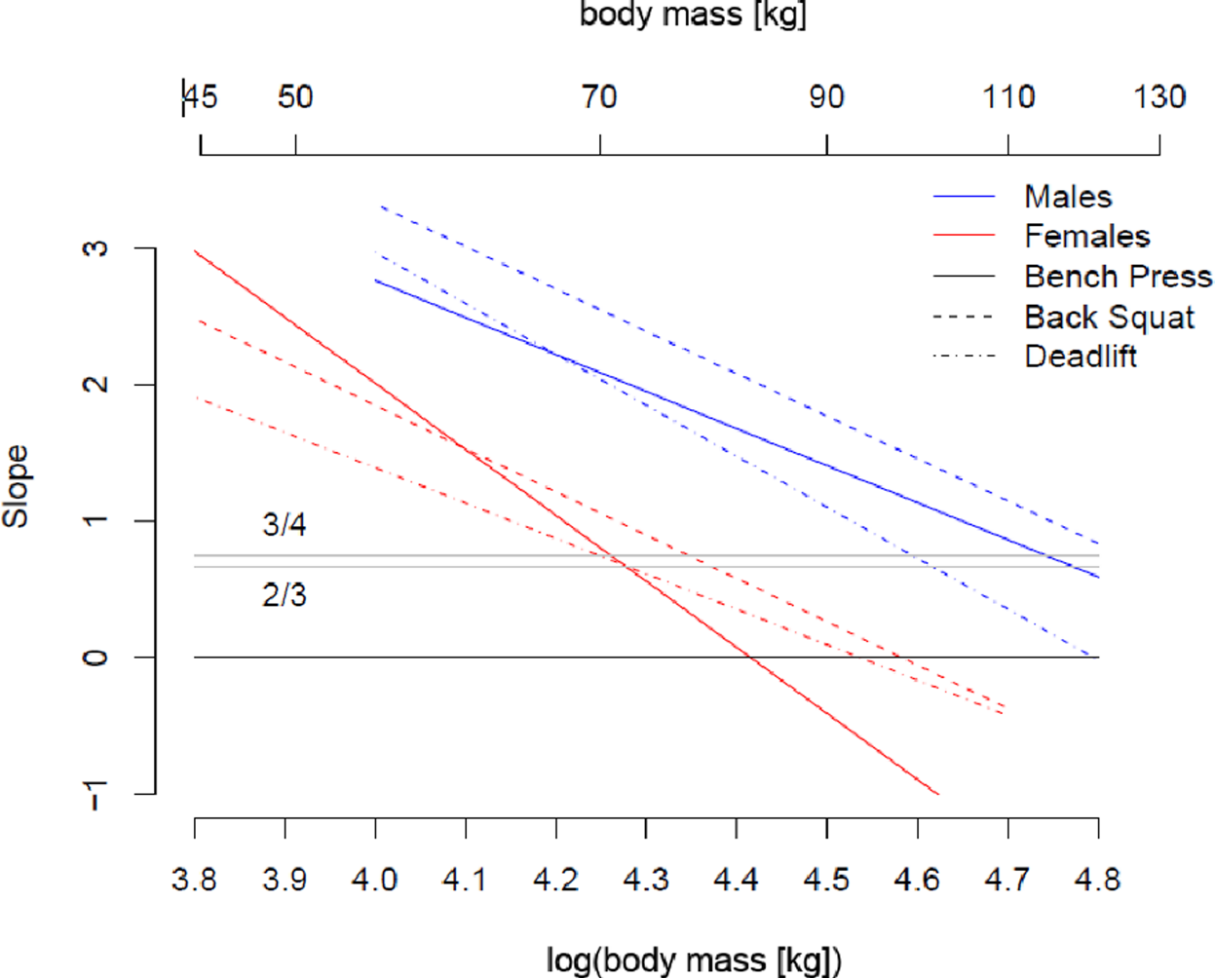
1 st derivatives of the curves presented in Fig. 1. Grey lines indicate conventional allometric scaling factors.

Figure 1 shows the curves resulting from the calculated relations of *log(1RM)* and *log(m).* All other independent variables have been stipulated to the respective medians. Figure 2 shows the corresponding 1 st derivatives (i.e. the slopes). The slopes for conventional allometric relations (Kleiber, 3/4^44^; Snell, 2/3^45^ are indicated by horizontal grey lines.

### Z-scores

**Fig. 3:**
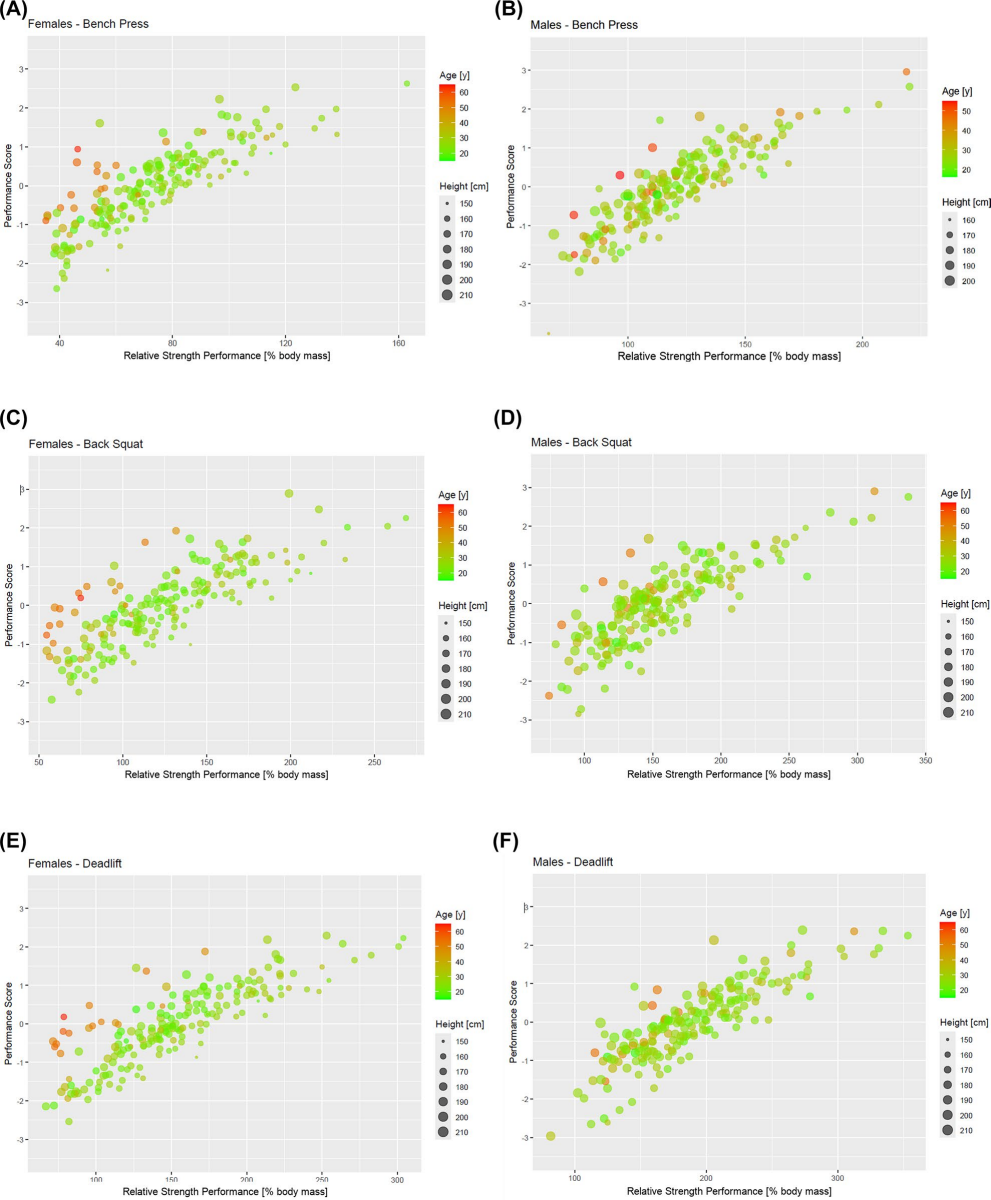
Z-scores in relation to relative strength performance. (**A**): Female bench press; (**B**): Male bench press; (**C**): Female back squat; (**D**): Male back squat; (**E**): Female deadlift; (**F**): Male deadlift.

Significant predictors differed between sexes and across exercises (BP, SQ, and DL), however, no meaningful differences were observed between the complete and consistently reduced models. Consequently, task-specific z-scores were calculated for all participants using the reduced model. These findings indicate that the reduced models sufficiently account for all relevant explanatory variables. Individual z-scores for each exercise are presented in Figs. 1A–F. Task-specific z-scores for individual participants can be obtained using the corresponding link or QR code provided. Figure 3.

Link: QR Code.

https://ist-hochschule.shinyapps.io/strengthindex/.


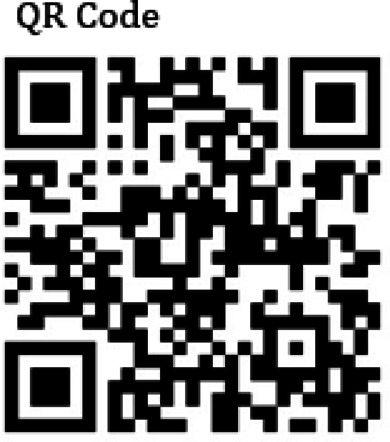


### Overlapping interval-based performance classification

For practical and interpretative purposes, an additional overlapping, interval-based performance classification system based on population-derived percentile ranges was applied. Seven performance levels were defined: untrained, beginner, intermediate, trained, advanced, highly advanced, and elite (Tables 3, 4, 5, 6, 7 and 8).

**Table 3:** Interval-based classification of z-score, percentile and 1-RM for female individuals in bench press.

Category	z-score range (± 0.1)	Percentile range (%)	1-RM absolute (kg)*	1-RM relative*
Untrained	< −1.4	0 to 10	30.8 ± 4.6	0.44 ± 0.06
Beginner	−1.6 to −0.4	7 to 36	36.4 ± 5.9	0.55 ± 0.10
Intermediate	−0.6 to 0.6	28 to 74	47.8 ± 9.0	0.73 ± 0.13
Trained	0.4 to 1.1	63 to 86	54.7 ± 10.6	0.83 ± 0.15
Advanced	0.9 to 1.6	85 to 95	68.0 ± 13.5	1.02 ± 0.21
Highly Advanced	1.4 to 2.1	93 to 98	79.6 ± 10.4	1.10 ± 0.23
Elite	> 1.9	98 to 99	90.5 ± 9.3	1.27 ± 0.25

*1-RM values do not take age and height into account; the average values are based on the z-score for ranking.

**Table 4:** Interval-based classification of z-score, percentile and 1-RM for male individuals in bench press.

Category	z-score range (± 0.1)	Percentile range (%)	1-RM absolute (kg)*	1-RM relative*
Untrained	< −1.4	0 to 8	71.9 ± 12.6	0.85 ± 0.10
Beginner	−1.6 to −0.4	7 to 34	85.4 ± 15.9	1.00 ± 0.12
Intermediate	−0.6 to 0.6	27 to 75	101.7 ± 18.9	1.20 ± 0.15
Trained	0.4 to 1.1	70 to 87	119.1 ± 20.4	1.39 ± 0.14
Advanced	0.9 to 1.6	84 to 95	127.4 ± 18.5	1.49 ± 0.14
Highly Advanced	1.4 to 2.1	93 to 98	132.8 ± 31.9	1.60 ± 0.23
Elite	> 1.9	97 to 99	161.2 ± 34.7	1.95 ± 0.21

*1-RM values do not take age and height into account; the average values are based on the z-score for ranking.

**Table 5:** Interval-based classification of z-score, percentile and 1-RM for female individuals in back squat.

Category	z-score range (± 0.1)	Percentile range (%)	1-RM absolute (kg)*	1-RM relative*
Untrained	< −1.4	0 to 10	51.0 ± 13.0	0.75 ± 0.13
Beginner	−1.6 to −0.4	5 to 36	63.4 ± 14.8	0.94 ± 0.22
Intermediate	−0.6 to 0.6	26 to 70	80.2 ± 18.1	1.19 ± 0.27
Trained	0.4 to 1.1	64 to 83	101.0 ± 20.4	1.56 ± 0.25
Advanced	0.9 to 1.6	77 to 93	108.5 ± 22.3	1.64 ± 0.32
Highly Advanced	1.4 to 2.1	91 to 96	110.8 ± 24.7	1.67 ± 0.54
Elite	> 1.9	96 to 99	176.0 ± 33.0	2.36 ± 0.33

*1-RM values do not take age and height into account; the average values are based on the z-score for ranking.

**Table 6:** Interval-based classification of z-score, percentile and 1-RM for male individuals in squat.

Category	z-score range (± 0.1)	Percentile range (%)	1-RM absolute (kg)*	1-RM relative*
Untrained	< −1.4	0 to 7	87.7 ± 26.2	1.04 ± 0.19
Beginner	−1.6 to −0.4	5 to 33	108.6 ± 28.0	1.24 ± 0.22
Intermediate	−0.6 to 0.6	26 to 74	126.8 ± 28.1	1.50 ± 0.24
Trained	0.4 to 1.1	67 to 87	159.7 ± 34.7	1.83 ± 0.28
Advanced	0.9 to 1.6	83 to 94	175.0 ± 40.4	2.02 ± 0.30
Highly Advanced	1.4 to 2.1	91 to 96	184.2 ± 41.2	2.19 ± 0.36
Elite	> 1.9	96 to 99	246.0 ± 46.6	3.00 ± 0.26

*1-RM values do not take age and height into account; the average values are based on the z-score for ranking.

**Table 7:** Interval-based classification of z-score, percentile and 1-RM for female individuals in deadlift.

Category	z-score range (± 0.1)	Percentile range (%)	1-RM absolute (kg)*	1-RM relative*
Untrained	< −1.4	0 to 10	64.5 ± 10.4	0.90 ± 0.14
Beginner	−1.6 to −0.4	8 to 33	78.7 ± 14.0	1.18 ± 0.23
Intermediate	−0.6 to 0.6	26 to 69	95.2 ± 18.9	1.45 ± 0.29
Trained	0.4 to 1.1	65 to 85	125.9 ± 18.8	1.90 ± 0.24
Advanced	0.9 to 1.6	82 to 92	133.2 ± 26.0	1.99 ± 0.39
Highly Advanced	1.4 to 2.1	92 to 96	157.2 ± 24.6	2.28 ± 0.52
Elite	> 1.9	96 to 99	190.0 ± 13.9	2.67 ± 0.37

*1-RM values do not take age and height into account; the average values are based on the z-score for ranking.

**Table 8:** Interval-based classification of z-score, percentile and 1-RM for male individuals in deadlift.

Category	z-score range (± 0.1)	Percentile range (%)	1-RM absolute (kg)*	1-RM relative*
Untrained	< −1.4	0 to 8	105.0 ± 13.0	1.21 ± 0.20
Beginner	−1.6 to −0.4	5 to 35	129.4 ± 23.9	1.51 ± 0.19
Intermediate	−0.6 to 0.6	27 to 73	156.1 ± 31.0	1.86 ± 0.29
Trained	0.4 to 1.1	65 to 86	190.2 ± 37.9	2.18 ± 0.27
Advanced	0.9 to 1.6	82 to 94	210.7 ± 38.5	2.38 ± 0.29
Highly Advanced	1.4 to 2.1	92 to 98	234.8 ± 51.1	2.75 ± 0.42
Elite	> 1.9	97 to 99	253.8 ± 52.3	2.97 ± 0.48

*1-RM values do not take age and height into account; the average values are based on the z-score for ranking.

### Multivariate evaluation

The multivariate evaluation was performed to identify atypical performance profiles across the assessed variables. Mahalanobis distances were calculated based on the combined distribution of the selected performance measures (BP, SQ, DL). Observations exceeding the χ² threshold were retained in the dataset but visually highlighted to facilitate interpretation of potential multivariate outliers.

**Fig. 4:**
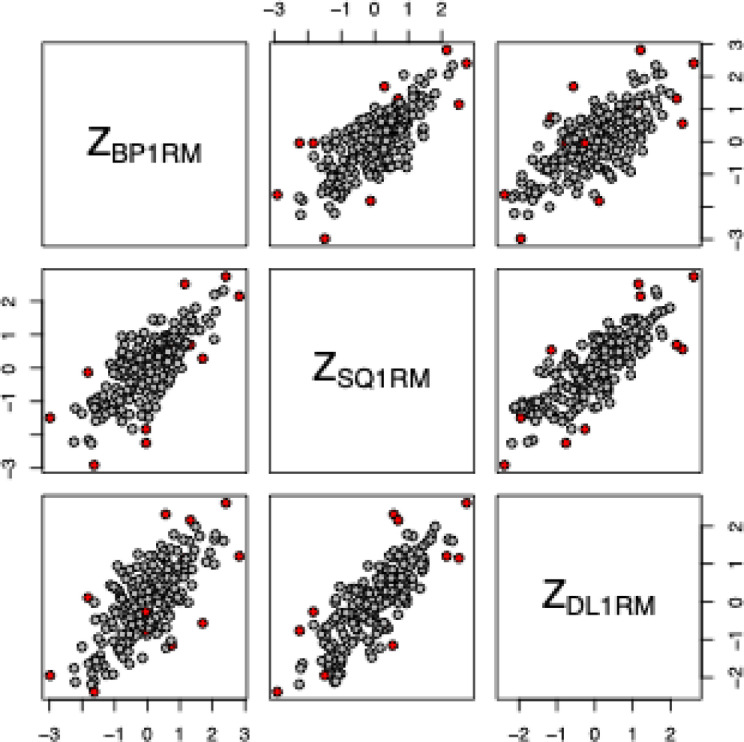
Scatterplot matrix of the performance scores of the female athletes. Red symbols indicate $\:{D}_{M}^{2}>7.81$.

**Fig. 5:**
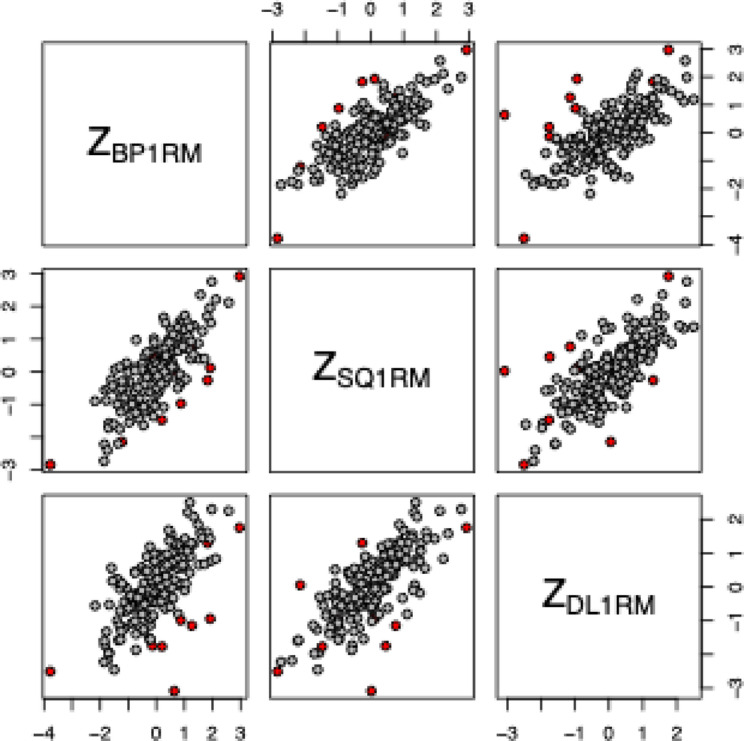
Scatterplot matrix of the performance scores of the male athletes. Red symbols indicate $\:{D}_{M}^{2}>7.81$.

Figures 4 and 5 present scatterplot matrices of performance scores for female and male athletes, respectively. Data points corresponding to Mahalanobis distances $\:{D}_{M}^{2}>7.81$ are highlighted in red, indicating potential “outliers”. This threshold of 7.81 represents the 95th percentile of the $\:{\chi\:}^{2}$-distribution with 3 degrees of freedom ($\:{\chi\:}_{3}^{2}$), identifying observations that deviate significantly from the expected performance distribution.

## Discussion

The primary aim of this study was to develop a standardized and empirically grounded framework for the assessment and classification of 1-RM performance in BP, SQ and DL among healthy individuals aged 16 to 64 years. Unlike conventional approaches that rely on absolute values or simple body mass-normalized measures, this approach explicitly accounts for inter-individual variability in anthropometric and demographic characteristics. By applying multiple regression and allometric scaling, raw strength data were transformed into standardized z-scores, providing a biologically meaningful interpretation of maximal strength capacity.

The present study systematically examined the contributions of age, body mass, and anthropometric parameters with notes of allometry to 1-RM performance using multiple regression analyses. The reduced predictive model retained only variables demonstrating significant and independent associations with performance outcomes, while achieving similar predictive accuracy to the complete model. This finding suggests that body mass, height, and age represent sufficient and practical predictors for individualized strength assessment in the BP, SQ and DL, without requiring more complex anthropometric measurements such as limb segment lengths. The resulting z-scores provide an objective, task-specific metric for classifying 1-RM performance while appropriately accounting for relevant anthropometric and age-related differences. The findings of the present study are consistent with and extend existing research on strength testing and performance assessment. Although 1-RM testing is widely regarded as the criterion standard for evaluating maximal dynamic strength^40,48–50^, both absolute 1-RM values and body mass normalized indices are limited in their ability to capture inter-individual differences attributable to anthropometry^18,33,39,51^. Furthermore, several currently applied strength classification frameworks are predominantly based on theoretical models rather than empirically derived reference data task-specific^34,52,53^. The present study addresses this gap by adopting a data-driven modelling approach that integrates measured anthropometric variables into the derivation of standardized performance scores. It is important to note, however, that absolute 1-RM values retain practical utility in contexts where absolute force output is the primary criterion, such as contact sports or occupational settings requiring load carriage (e.g., firefighting). The proposed framework is therefore intended to complement, rather than replace, absolute strength measures^54^.

Our findings consistently demonstrate that strength performance exhibits a nonlinear allometric relationship with body mass that is not fully captured by a simple power-law model. The observed curvature indicates that the allometric association varies systematically across the body mass spectrum, suggesting the need for second-order, and potentially higher-order, terms on the right-hand side of Eq2. This interpretation is consistently supported by the partial residual plots for both log(m) and log(m)^2^ across all combinations of sex, exercise, and model type (see Supplementary Material). Importantly, the derivatives of the fitted curves (Fig. 2) further illustrate that the effective scaling exponent is not constant across body mass. Rather than converging on the conventional allometric coefficients of 2/3 or 3/4, the estimated local exponents generally fall outside these values and only approximate them within narrow ranges of body mass. These findings suggest that maximal strength performance is not adequately described by the assumptions underlying classical allometric models. Whereas traditional allometric theory was largely developed to explain biological processes governed by geometric constraints and steady substrate fluxes, 1-RM performance is influenced by a broader set of determinants, including morphological, neuromuscular, and training-related factors. Changes in neuromuscular interaction patterns with increasing muscle mass may therefore contribute to the observed nonlinear scaling behavior, and these mechanisms are likely to differ between sexes and potentially across muscle groups. Consequently, the common practice of normalizing strength measures using a fixed exponent such as m^3/4^ should be critically reconsidered as it may introduce systematic bias when comparing individuals across a broad range of body masses. Instead, the present findings support the use of models incorporating both log(m) and log(m)^2^, which more appropriately capture the nonlinear nature of the body mass–strength relationship and may therefore provide a more robust basis for anthropometry-adjusted strength assessment. From a conceptual perspective, this approach draws on principles established in reference-value frameworks in exercise physiology, in which performance indicators are routinely normalized to relevant covariates to enhance interpretability across individuals^29,55,56^. By introducing anthropometry-adjusted strength metrics and standardized z-scores, the present study establishes an analogous diagnostic framework for resistance training. Critically, this approach enables more effective tracking of individual strength changes over time, improves applicability in sport performance monitoring, and supports health-related strength assessment across individuals of differing body dimensions and age profiles.

However, when strength scores are implemented within ordinal scaling systems or overlapping interval–based classification frameworks, the results demonstrate that key determinants of performance, such as age or body height, cannot be adequately accounted for. Consequently, substantial variability within assigned performance categories may occur, leading to wide and heterogeneous ranges of actual strength capacity within the same classification level. This limitation is not unique to the present study; similar heterogeneity within performance categories has been reported in classification frameworks applied in both endurance and strength-oriented assessments^30,34,57^.

Given the multidimensional nature of factors influencing physical performance, the use of ordinal scaling models, currently prevalent in both strength and endurance testing^30,34,57^, should be interpreted with caution and, where possible, avoided. Instead, in line with established practices in physiological assessment^28,58^, performance evaluation should be based on multifactorial scoring approaches that integrate relevant covariates and reference empirically derived distributions from representative samples. Such an approach allows a more precise, individualized, and physiologically meaningful classification of performance capacity.

The large and diverse sample size of this study (*n* = 393; S1–S3) represents a major methodological strength, enabling robust statistical modeling across a wide range of performance levels, ages, and body compositions. The application of multiple regression, allometric transformations, and Mahalanobis distance calculations provides a rigorous, data-driven basis for strength classification and outlier identification. These methods allow contextualisation of an individual’s strength capacity relative to others with comparable anthropometric and demographic profiles, which may support performance monitoring and training programme individualisation. The framework is, however, limited to cross-sectional interpretation and does not permit causal inferences or conclusions regarding longitudinal performance development.

The proposed framework facilitates individualized comparison of 1-RM performance by accounting for sex, age, body mass, and height. For meaningful application, it is essential that these demographic and anthropometric characteristics are reported alongside strength values in research and applied settings. Incorporating additional anthropometric factors such as arm or thigh length may further refine classification, although the present results indicate that body height alone can serve as an effective proxy in the reduced model. By accounting for these variables, the load required to achieve specific performance levels can be estimated a priori, which may improve study design and facilitate more accurate participant classification in future research.

Accurate determination of individual performance capacity remains inherently limited by unmeasured physiological variables, including muscle fiber composition^59,60^ and motor unit recruitment patterns^61,62^. Techniques such as muscle biopsies^63,64^, electromyography (EMG)^65^, and electroencephalography (EEG)^66^ could provide more detailed insights but are often impractical for large-scale or applied settings. In contrast, incorporating readily measurable variables such as age^67^, sex^68^, anthropometric measures, and body mass represents a feasible and practical strategy for achieving a reliable and meaningful assessment of 1-RM^18,37,38,69,70^.

### Practical implications

The framework has both scientific and applied utility. From a scientific perspective, the z-score approach enables retrospective comparability across studies: when age, body mass, and height are reported alongside 1-RM values, the relative strength levels of samples from different studies can be meaningfully compared regardless of differences in participant characteristics. Prospectively, the framework supports study design by allowing researchers to define target performance levels or stratify participants by strength capacity independent of anthropometric characteristics, thereby improving the precision of participant classification in future investigations.

### Limitations and future research directions

While this study presents a novel approach to strength classification through anthropometric scaling and statistical modeling, several limitations must be acknowledged. First, the analysis focused exclusively on 3 resistance exercises (BP, SQ, and DL), which, although widely used, may not fully capture all dimensions of muscular strength. Inclusion of additional compound or isolated movements, such as Olympic lifts or isometric strength assessments, could provide further insights into sport-specific strength profiles.

Second, despite adherence to strict NSCA protocols, standardization challenges in strength testing remain. Minor variations in bar path, squat depth, or execution technique (bridge or grip width) may have influenced 1-RM outcomes. Future research should therefore investigate the effects of movement variability and explore strategies for real-time biomechanical standardization in strength assessments.

Third, the derived scoring system is applicable only to the representative population studied, namely healthy individuals aged 16–64 years. Its validity and interpretability for younger or older individuals remain unknown. Furthermore, due to the technical complexity of the examined lifts, the framework cannot be reliably applied to other populations with markedly different physical capacities, limiting generalizability. Accordingly, conclusions should be confined to 1-RM in the BP, SQ, and DL within this population. Furthermore, training status, competitive experience, and neuromuscular adaptations were not formally recorded as continuous covariates and may contribute to residual variance not accounted for by the anthropometric predictors.

Future research should examine the applicability of this framework to populations beyond the age range of 16–64 years and to exercises other than the SQ, BP and DL. Additionally, studies should investigate the framework’s sensitivity to longitudinal performance changes, as this would provide valuable information for tracking training adaptations over time. The inclusion of training status and competitive experience as covariates in future models would further improve predictive accuracy and classification reliability.

## Conclusion

This study presents a standardized, anthropometry-based framework that advances strength testing beyond simple ratios. By integrating multiple regression modeling, allometric scaling, and z-score calculations, the framework accounts for individual biomechanical and physiological differences and enables the identification of outliers and exceptional performers. For healthy adults aged 16–64 and focusing on BP, SQ, and DL, this framework enables a more objective and individualized evaluation of maximal strength, facilitating better training optimization and enhanced research comparability.

## Supplementary Information

Below is the link to the electronic supplementary material.

Supplementary Material 1

## Data Availability

The datasets generated during and/or analyzed during the current study are available from the corresponding author on reasonable request.
