# Supplementary material for: Normalization of muscle strength measures in barbell exercises by sex, age, and anthropometry: notes on allometry
Source: Sci Rep. 2026 Jul 6;16:20797. doi: 10.1038/s41598-026-60646-z (PMC13338387; doi:10.1038/s41598-026-60646-z)

Supplemental Material:

S1: Table: Age range of participants divided into decades. Absolute amount and percentage distribution

| Age [years] | <20 | < 30 | < 40 | < 50 | > 50 |
| --- | --- | --- | --- | --- | --- |
| Numbers of participants | 32 | 250 | 72 | 22 | 17 |
| Percentage | 8.1 | 63.6 | 18.3 | 5.6 | 4.3 |

S2: Table: Height of the participants divided into decades. Absolute amount and percentage distribution

| Height [cm] | <160 | < 170 | < 180 | < 190 | >190 |
| --- | --- | --- | --- | --- | --- |
| Numbers of participants | 23 | 101 | 146 | 102 | 21 |
| Percentage | 5.9 | 25.7 | 37.2 | 25.9 | 5.3 |

S3: Table: Body mass of the participants divided into decades. Absolute amount and percentage distribution

| Body mass [kg] | <60 | < 70 | < 80 | < 90 | < 100 | >100 |
| --- | --- | --- | --- | --- | --- | --- |
| Numbers of participants | 39 | 97 | 102 | 91 | 40 | 24 |
| Percentage | 9.9 | 24.7 | 25.9 | 23.2 | 10.2 | 6.1 |


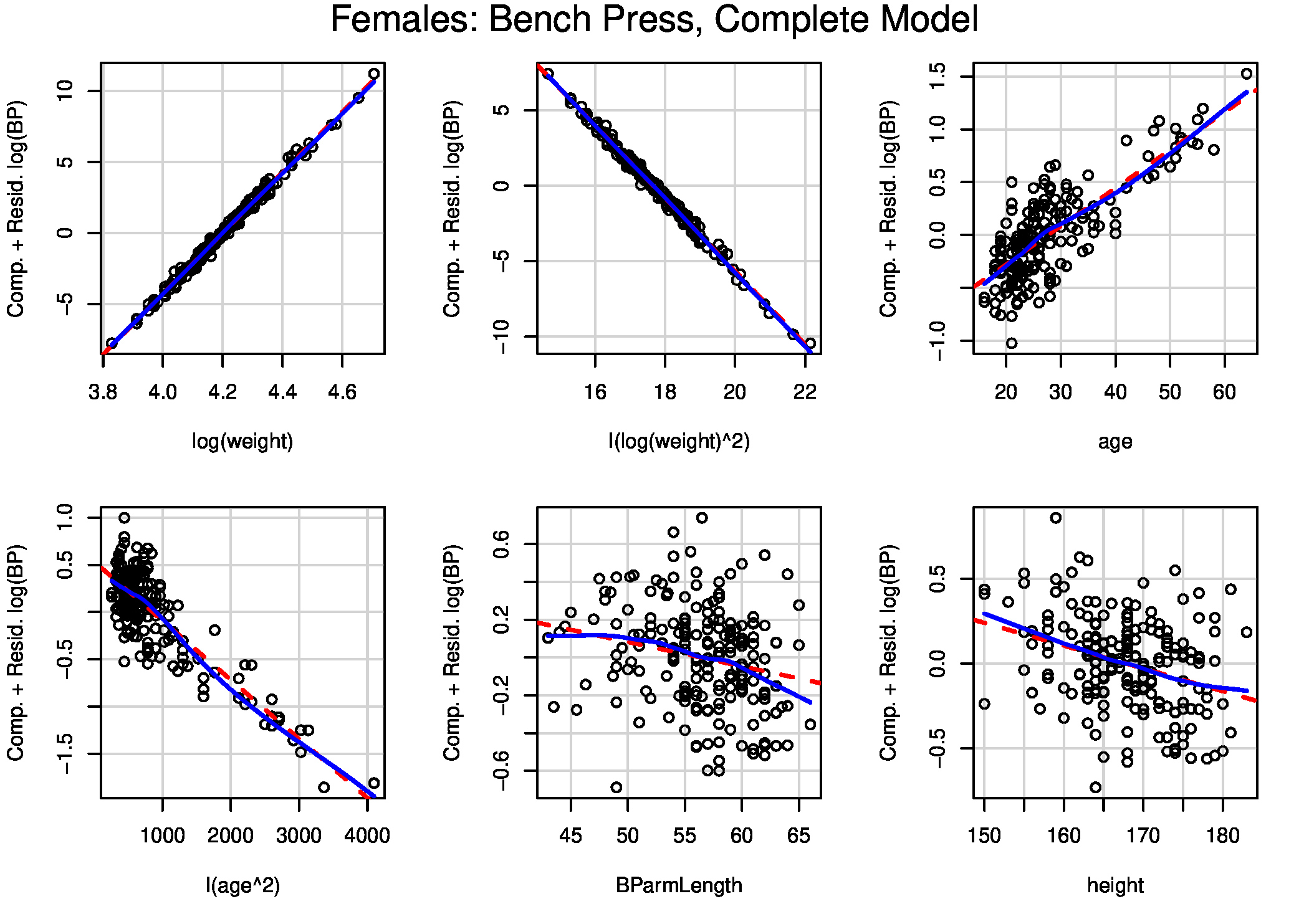


Figure S4 : Partial residual plots of the complete allometric model fitted to the performance levels of BP 1RM of female athletes.


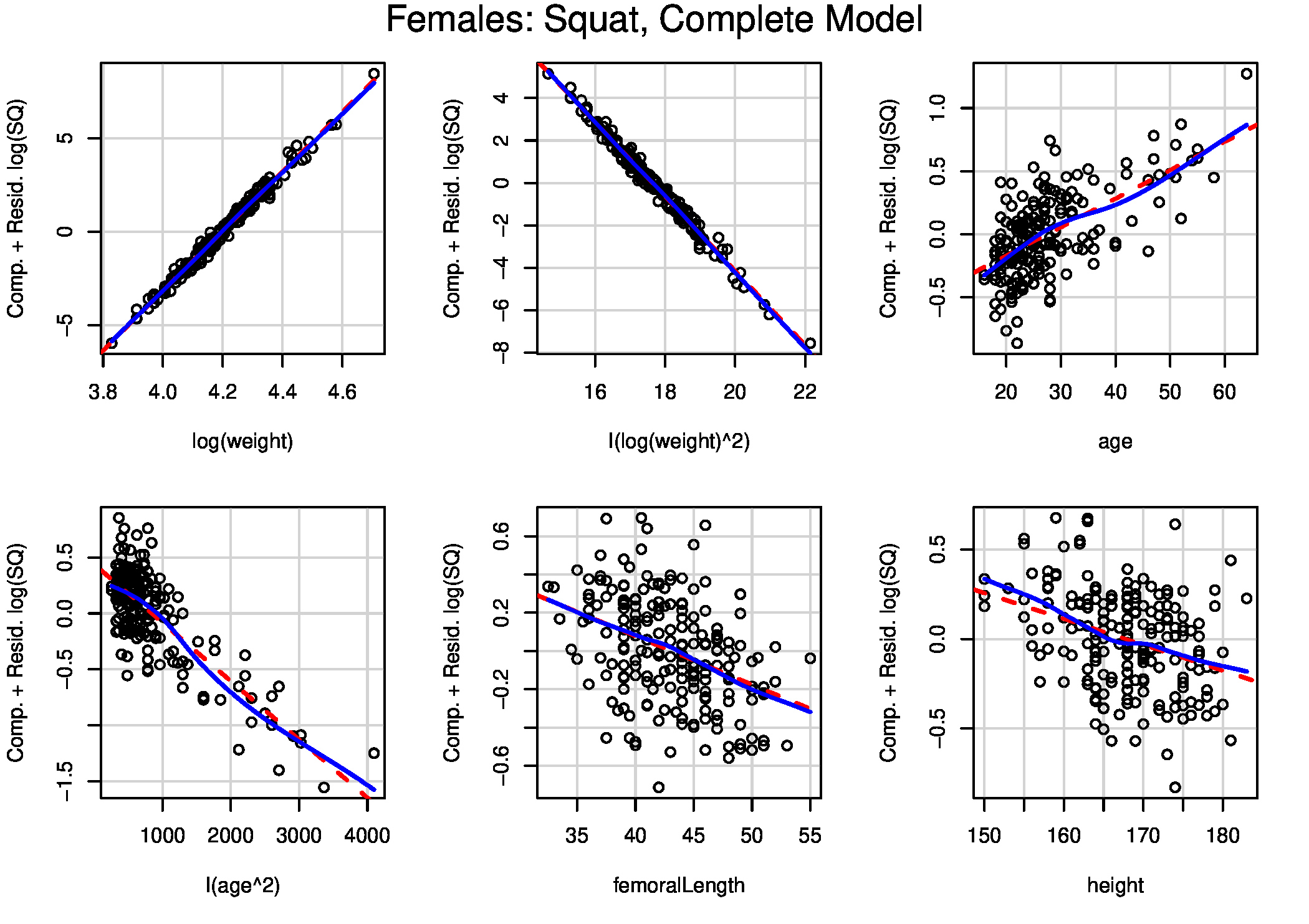


Figure S5: Partial residual plots of the complete allometric model fitted to the performance levels of SQ 1RM of female athletes


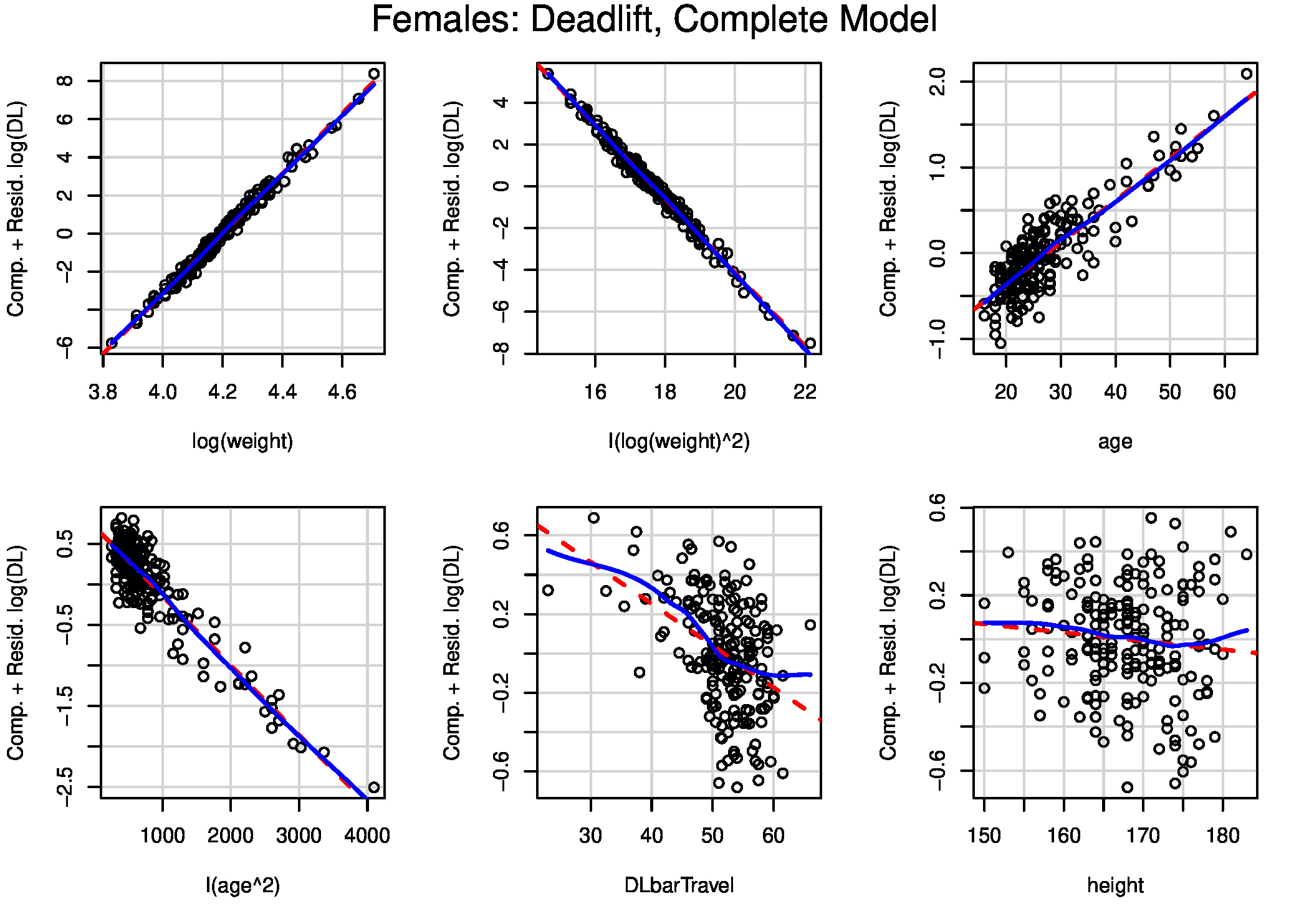


Figure S6:Partial residual plots of the complete allometric model fitted to the performance levels of DL 1RM of female athletes


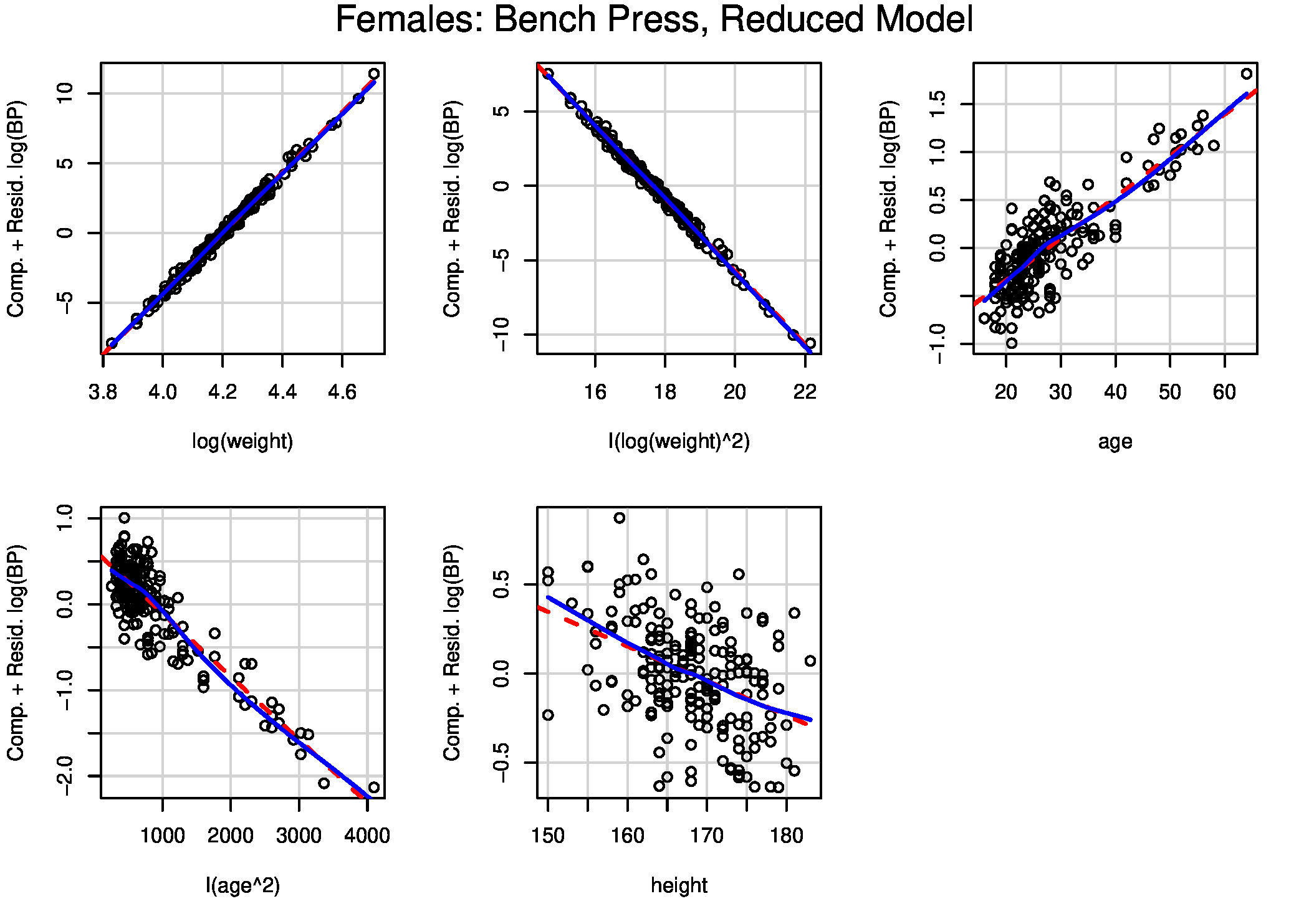


Figure S7: Partial residual plots of the reduced allometric model fitted to the performance levels of BP 1RM of female athletes


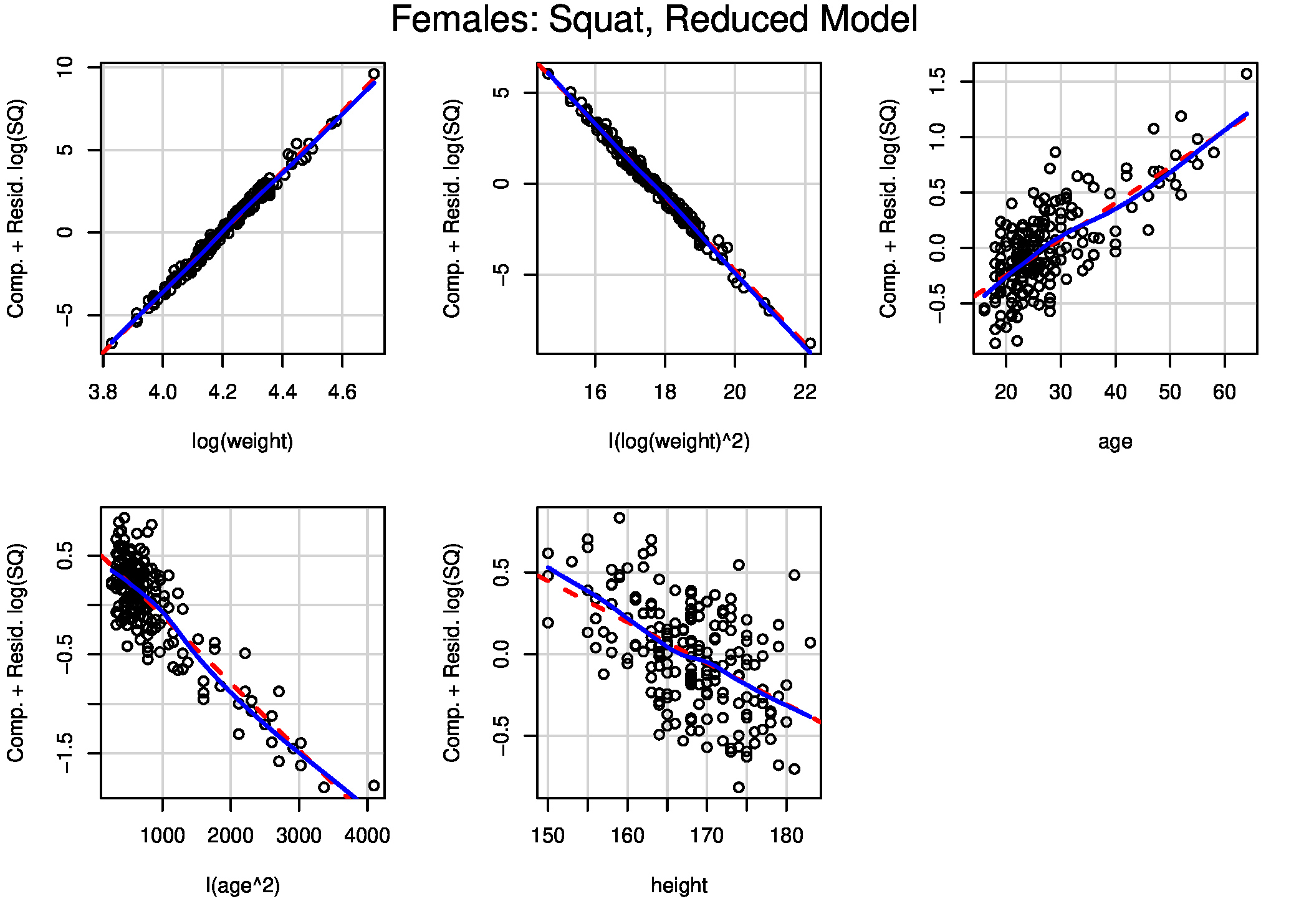


Figure S8: Partial residual plots of the reduced allometric model fitted to the performance levels of SQ 1RM of female athletes


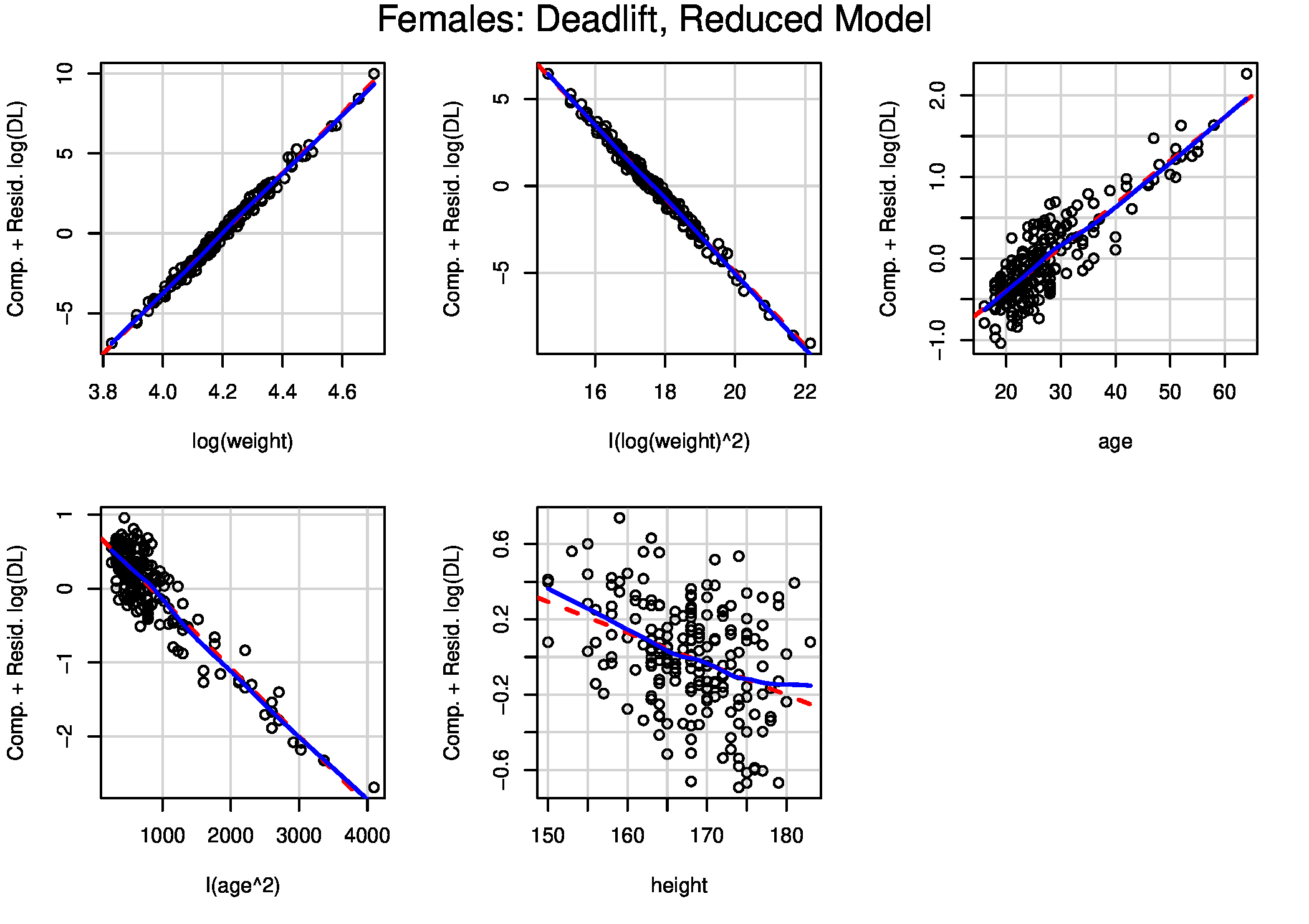


Figure S9: Partial residual plots of the reduced allometric model fitted to the performance levels of DL 1RM of female athletes


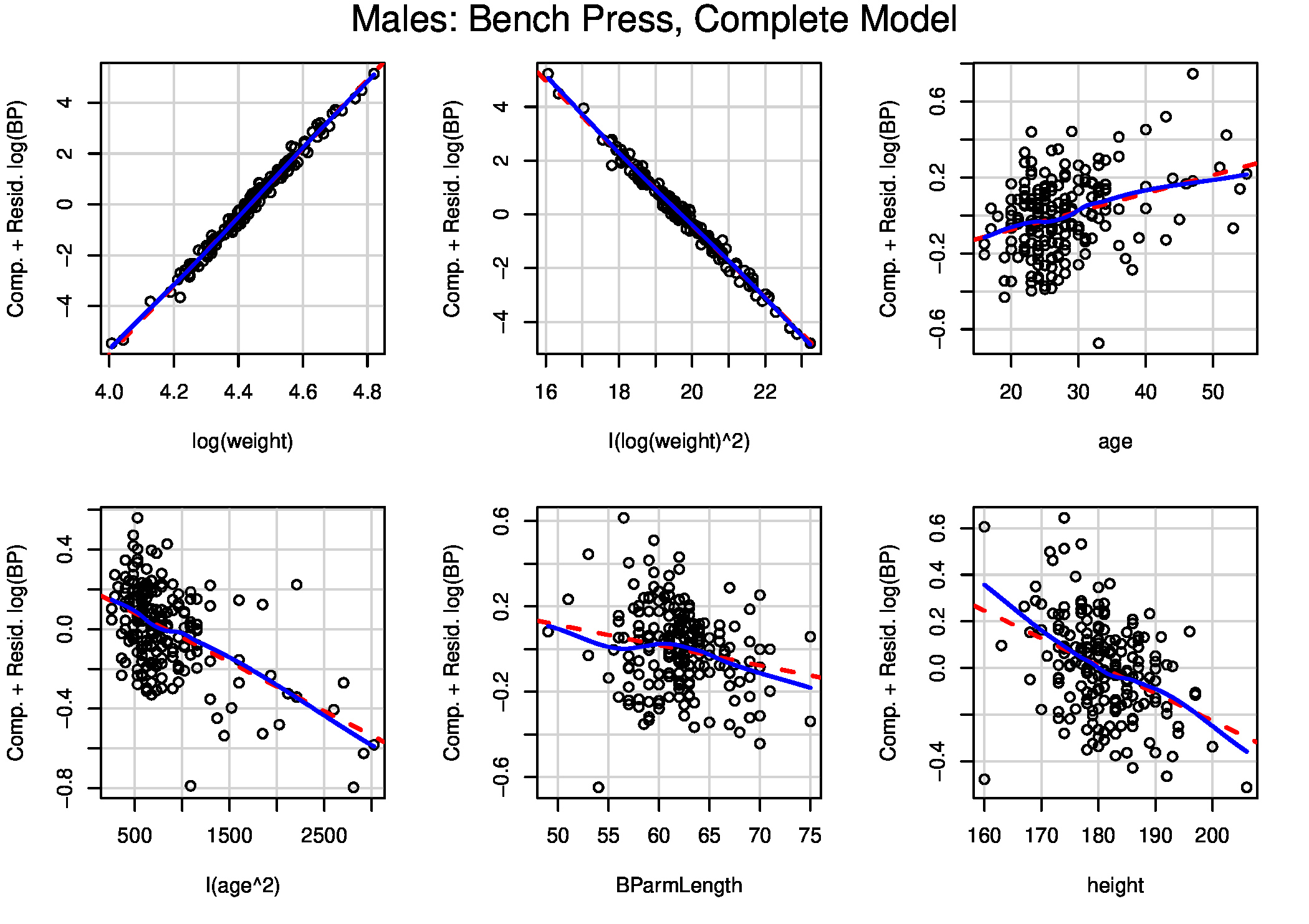


Figure S10: Partial residual plots of the complete allometric model fitted to the performance levels of BP 1RM of male athletes


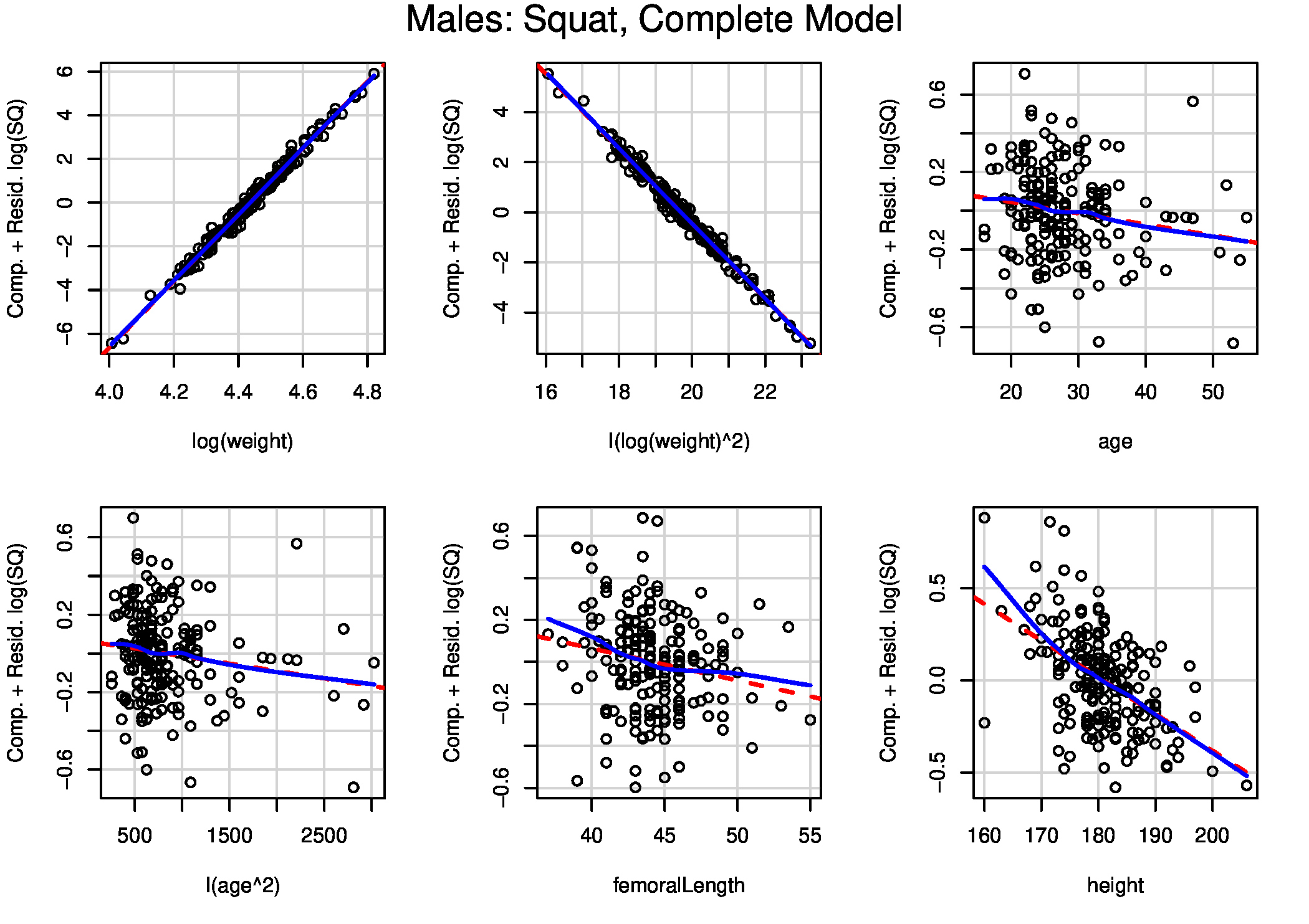


Figure S11: Partial residual plots of the complete allometric model fitted to the performance levels of SQ 1RM of male athletes


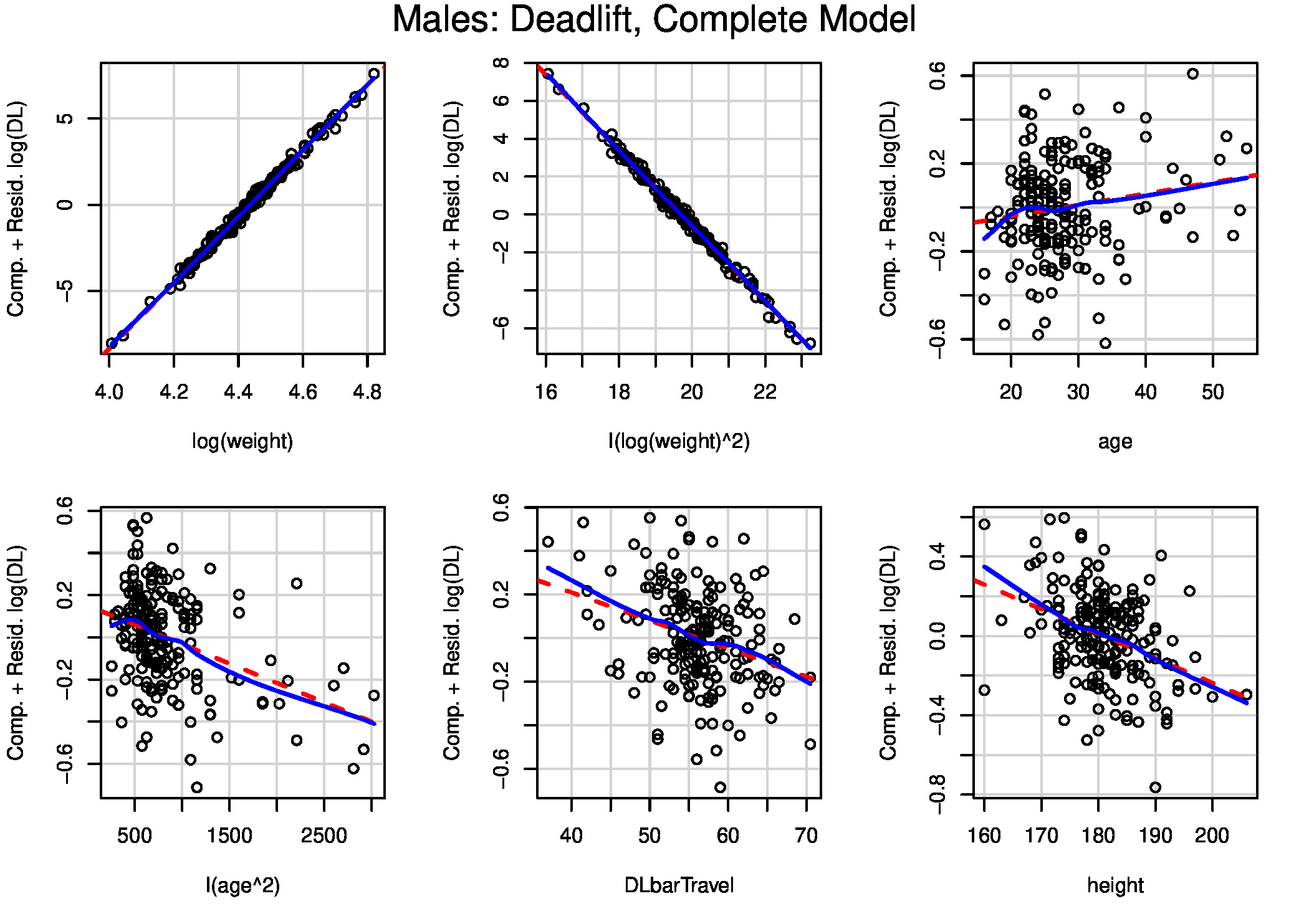


Figure S12: Partial residual plots of the complete allometric model fitted to the performance levels of DL 1RM of male athletes


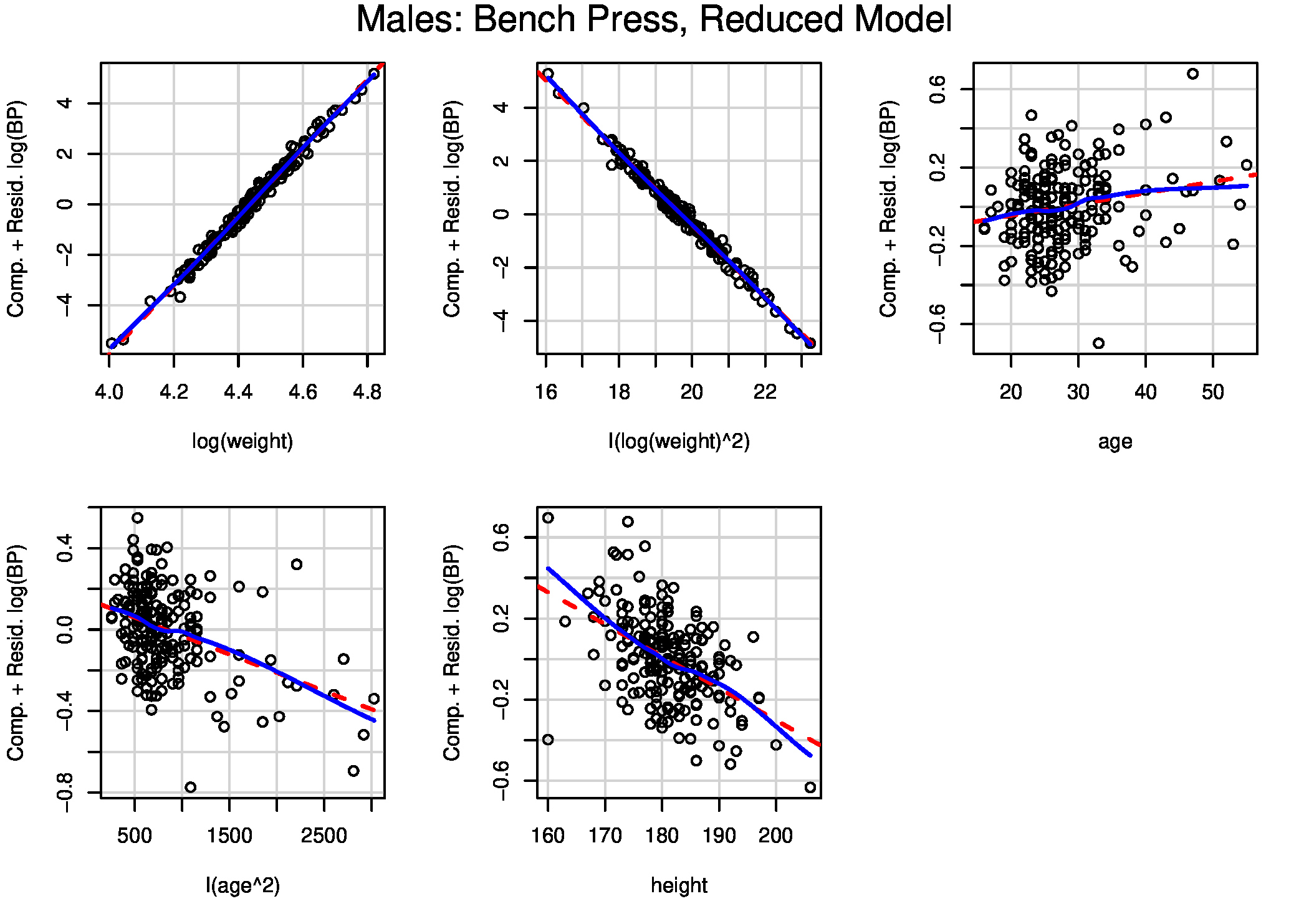


Figure S13: Partial residual plots of the reduced allometric model fitted to the performance levels of BP 1RM of male athletes


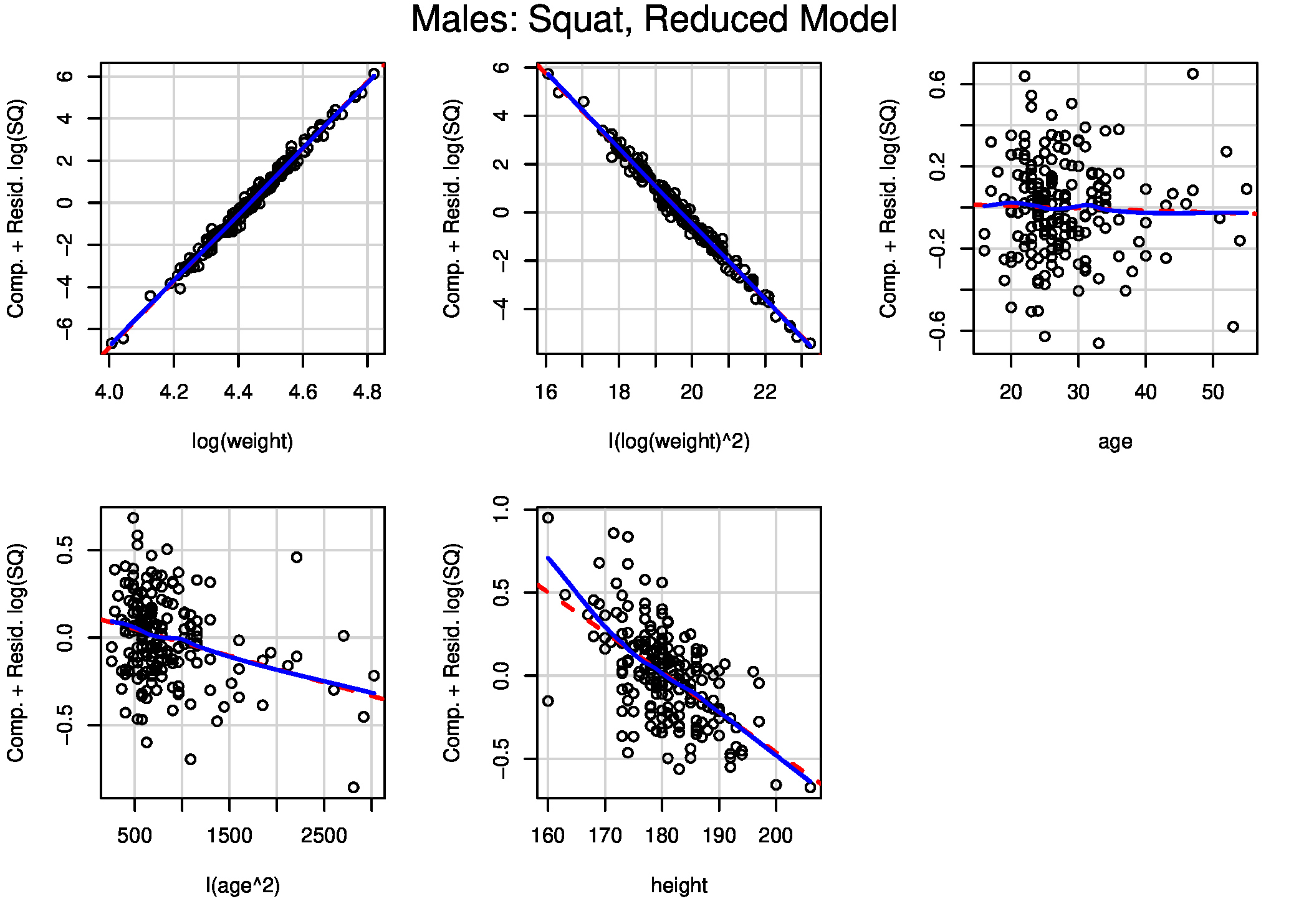


Figure S14: Partial residual plots of the reduced allometric model fitted to the performance levels of SQ 1RM of male athletes


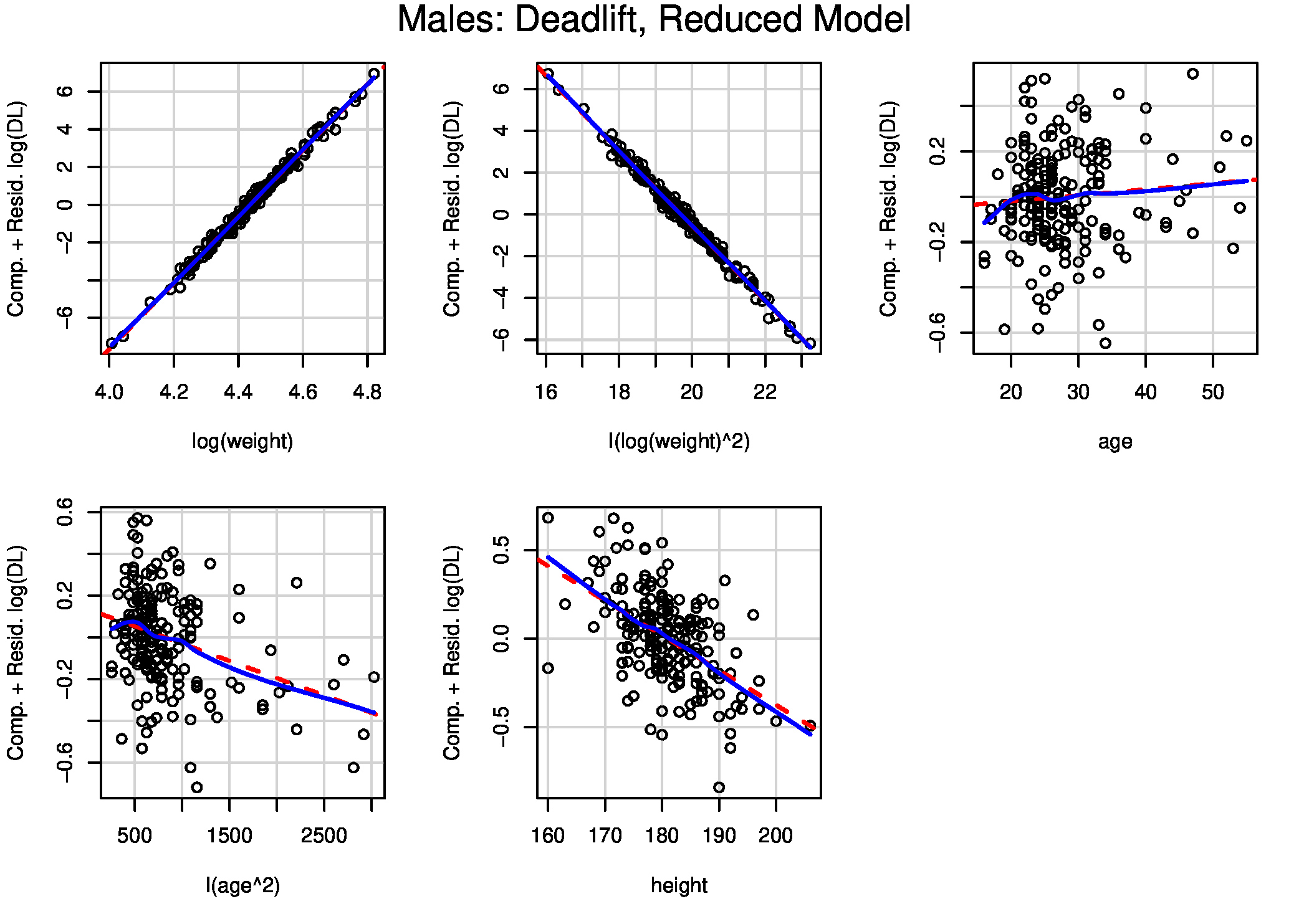


Figure S15: Partial residual plots of the reduced allometric model fitted to the performance levels of DL 1RM of male athletes

S16:Table:Residual standard deviations of the complete and reduced models fitted to the performances of female athletes.

|  | BP1RM | SQ1RM | DL1RM |
| --- | --- | --- | --- |
| **Complete** | 0.263 | 0.257 | 0.228 |
| **Reduced** | 0.267 | 0.276 | 0.269 |

S17:Table:Residual standard deviations of the complete and reduced models fitted to the performances of male athletes.

|  | BP1RM | SQ1RM | DL1RM |
| --- | --- | --- | --- |
| **Complete** | 0.193 | 0.230 | 0.218 |
| **Reduced** | 0.193 | 0.230 | 0.219 |

Figure S18: Ordinal scale of bench press performance - female


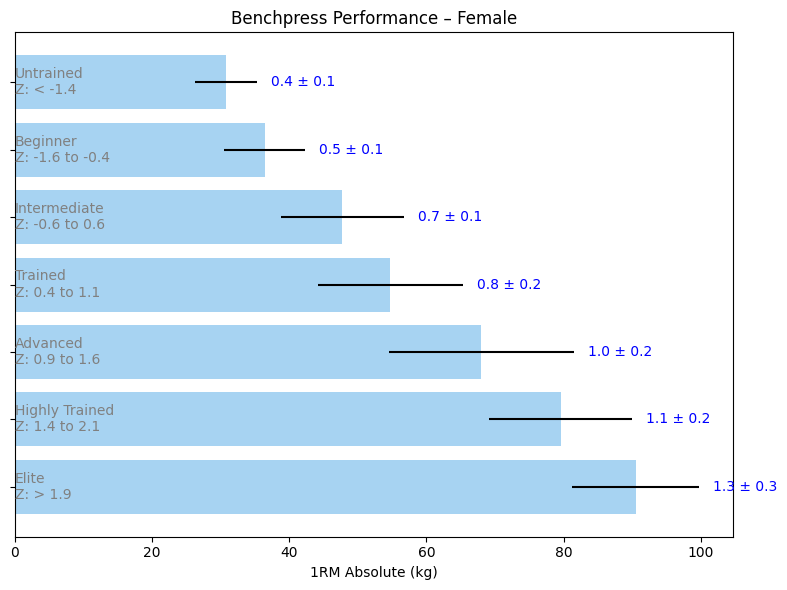


Z: z score range; blue values: relative strength; blue bars: absolute strength

Figure S19: Ordinal scale of squat performance - male


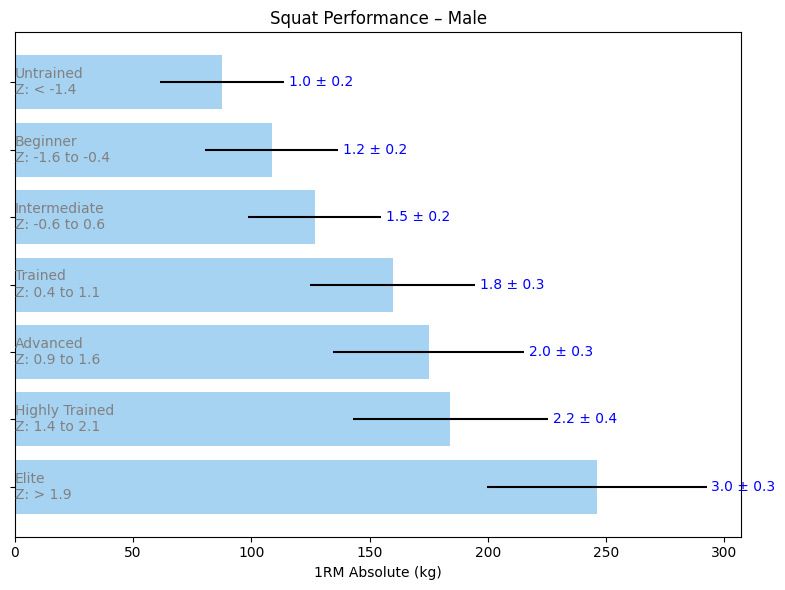


Figure S20: Ordinal scale of squat performance - female


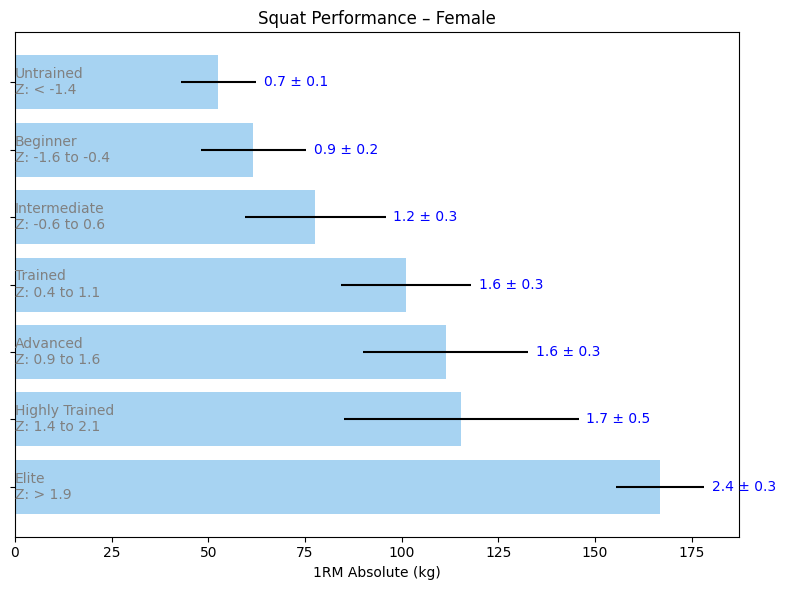


Figure S21: Ordinal scale of deadlift performance - male


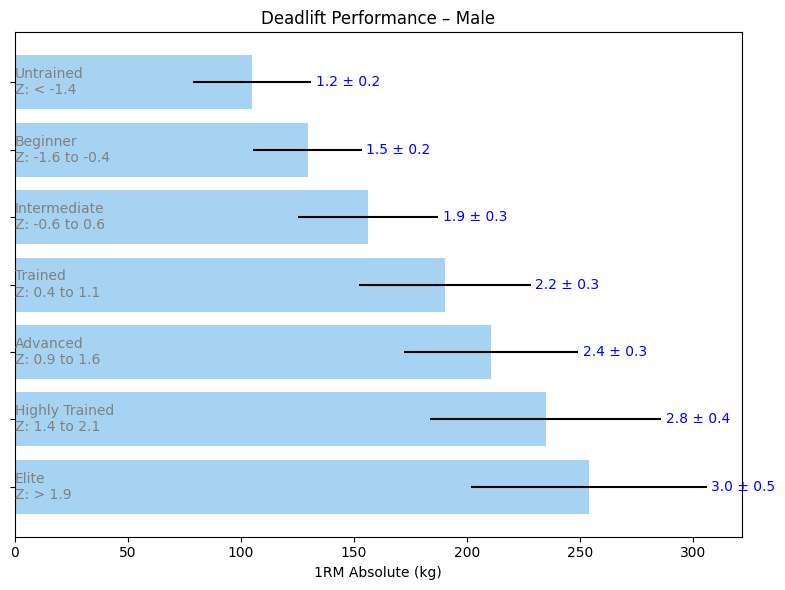


Figure S22: Ordinal scale of deadlift performance - female


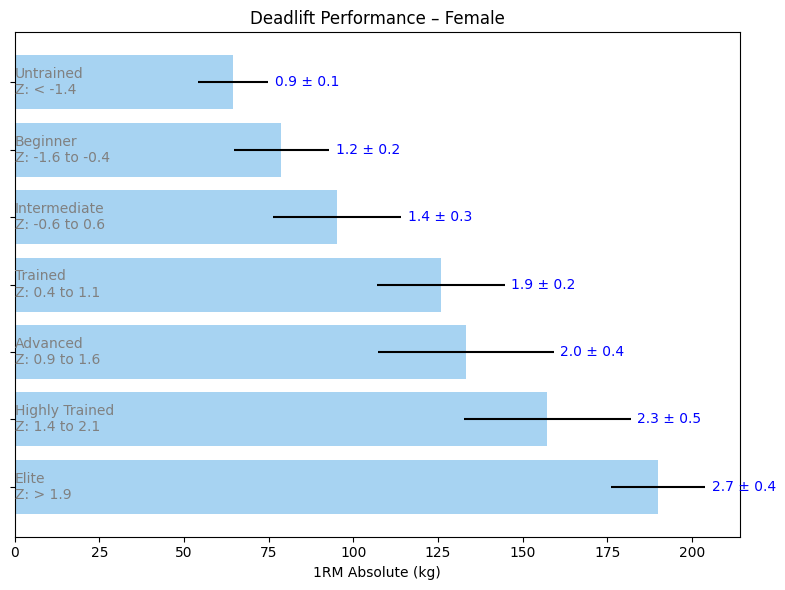


Figure S23: Ordinal scale of bench press performance - male


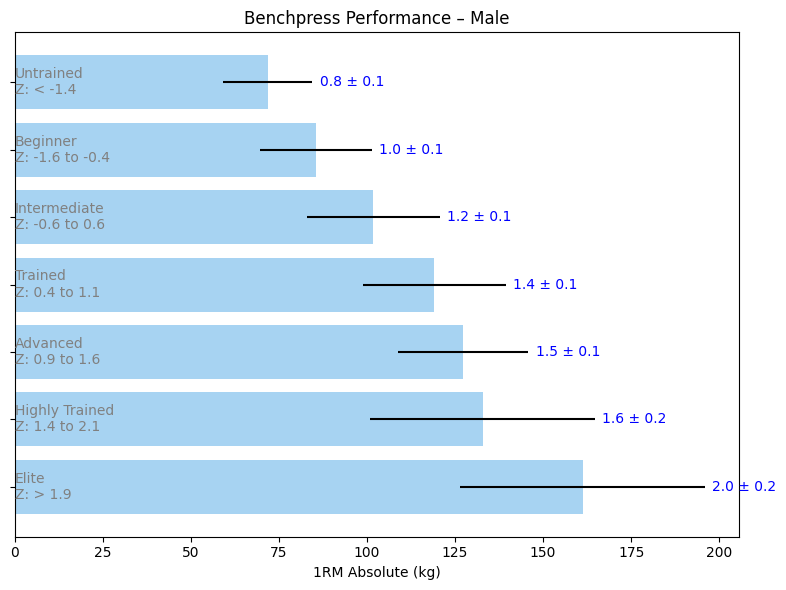


Figure S24: Ordinal scale of deadlift performance - female


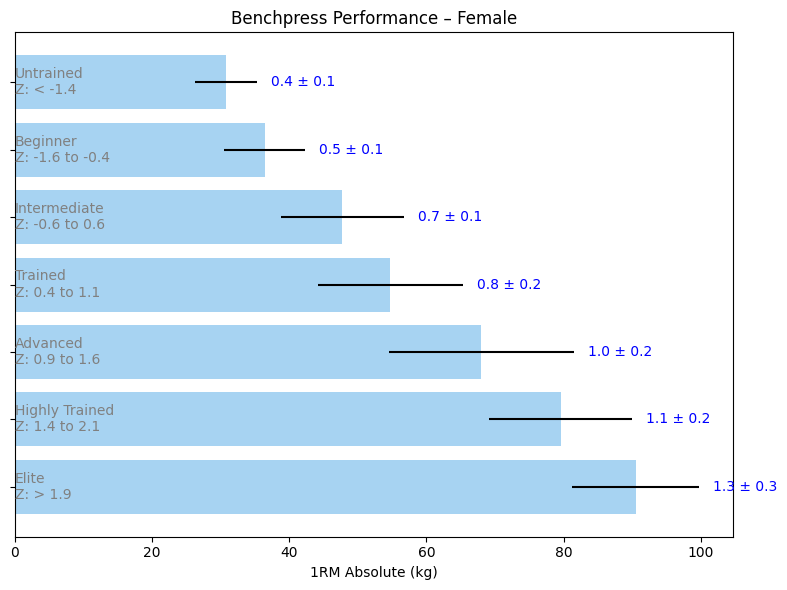

Supplement: Supplementary file 1 — Supplementary Material 1 [file 41598_2026_60646_MOESM1_ESM.docx]
